# Downregulation of Blood-Brain Barrier Phenotype by Proinflammatory Cytokines Involves NADPH Oxidase-Dependent ROS Generation: Consequences for Interendothelial Adherens and Tight Junctions

**DOI:** 10.1371/journal.pone.0101815

**Published:** 2014-07-03

**Authors:** Keith D. Rochfort, Laura E. Collins, Ronan P. Murphy, Philip M. Cummins

**Affiliations:** 1 School of Biotechnology, Dublin City University, Dublin, Ireland; 2 School of Health and Human Performance, Dublin City University, Dublin, Ireland; 3 Centre for Preventive Medicine, Dublin City University, Dublin, Ireland; Emory University School of Medicine, United States of America

## Abstract

**Background and Objectives:**

Blood-brain barrier (BBB) dysfunction is an integral feature of neurological disorders and involves the action of multiple proinflammatory cytokines on the microvascular endothelial cells lining cerebral capillaries. There is still however, considerable ambiguity throughout the scientific literature regarding the mechanistic role(s) of cytokines in this context, thereby warranting a comprehensive *in vitro* investigation into how different cytokines may cause dysregulation of adherens and tight junctions leading to BBB permeabilization.

**Methods:**

The present study employs human brain microvascular endothelial cells (HBMvECs) to compare/contrast the effects of TNF-α and IL-6 on BBB characteristics ranging from the expression of interendothelial junction proteins (VE-cadherin, occludin and claudin-5) to endothelial monolayer permeability. The contribution of cytokine-induced NADPH oxidase activation to altered barrier phenotype was also investigated.

**Results:**

In response to treatment with either TNF-α or IL-6 (0–100 ng/ml, 0–24 hrs), our studies consistently demonstrated significant dose- and time-dependent decreases in the expression of all interendothelial junction proteins examined, in parallel with dose- and time-dependent increases in ROS generation and HBMvEC permeability. Increased expression and co-association of gp91 and p47, pivotal NADPH oxidase subunits, was also observed in response to either cytokine. Finally, cytokine-dependent effects on junctional protein expression, ROS generation and endothelial permeability could all be attenuated to a comparable extent using a range of antioxidant strategies, which included ROS depleting agents (superoxide dismutase, catalase, *N*-acetylcysteine, apocynin) and targeted NADPH oxidase blockade (gp91 and p47 siRNA, NSC23766).

**Conclusion:**

A timely and wide-ranging investigation comparing the permeabilizing actions of TNF-α and IL-6 in HBMvECs is presented, in which we demonstrate how *either* cytokine can similarly downregulate the expression of interendothelial adherens and tight junction proteins leading to elevation of paracellular permeability. The cytokine-dependent activation of NADPH oxidase leading to ROS generation was also confirmed to be responsible in-part for these events.

## Introduction

Diminished blood-brain barrier (BBB) function is an integral feature of neurological disorders such as stroke [1], neurodegenerative diseases [2], traumatic brain injury [3] and neural infections [4]. A likely correlated aspect of the BBB breakdown associated with these pathologies is the production and release of multiple classes of proinflammatory cytokines from cells within the neurovascular unit and periphery (e.g. immune cells), which may cause barrier dysfunction of the microvascular endothelial cells lining the lumen of cerebral capillaries. Cytokines such as tumour necrosis factor-α (TNF-α) for example, have been strongly linked to neurological disorders [5], [6], whilst several studies have confirmed the ability of TNF-α to increase the permeability of brain microvascular endothelial cells [7]–[10]. Despite this, there is still much that is unclear regarding the cytokine-dependent mechanisms underlying permeabilization of the paracellular pathway across the BBB endothelium. Whilst some studies indicate that cytokines may alter the expression and/or distribution of interendothelial junction proteins, there are several gaps and inconsistencies within the existing knowledge base. This is evidenced by a noticeable scarcity of cytokine dose- and time-dependency studies, cytokine cross-comparative studies, and mechanistic signaling details in relevant BBB models. Highly variable findings across different endothelium models (both peripheral and cerebrovascular) is also manifest within the literature [11]–[13], leading to disparate and limited conclusions. As such, a definitive and comprehensive *in vitro* investigation into how functionally distinct proinflammatory cytokines may impact adherens junction (AJ) and tight junction (TJ) protein dynamics within a pertinent human BBB microvascular endothelial model is warranted.

The present study employs primary-derived human brain microvascular endothelial cells (HBMvECs) to compare/contrast the effects of TNF-α and interleukin-6 (IL-6), proinflammatory cytokines that act through distinct intracellular signalling pathways, on the expression of the interendothelial junction proteins VE-cadherin (AJ), occludin (TJ) and claudin-5 (TJ), in parallel with their effects on HBMvEC monolayer permeability. The three aforementioned protein targets are central to proper maintenance of paracellular permeability and so provide a reliable readout of BBB integrity. Complexation of the latter transmembrane TJ proteins with cytoplasmic adaptors such as zonnula occludens-1 (ZO-1) for example, create highly polarized paracellular barriers with selective permeability to water and solutes [14], whilst AJ-associated VE-cadherin has been shown to regulate TJ complexation and to stabilize BBB function [15]. As neurological disorders frequently manifest elevated levels of reactive oxygen species (ROS), deriving in-part from the cytokine-dependent activation of endothelial NADPH oxidase [16]–[18], the contribution of cytokine-induced NADPH oxidase activation and ROS generation to HBMvEC barrier properties was also investigated. In response to treatment with *either* TNF-α or IL-6, our studies consistently demonstrate significant dose- and time-dependent decreases in the expression of all AJ and TJ proteins examined (mRNA and protein), in parallel with dose- and time-dependent increases in HBMvEC permeability and ROS production. Importantly, cytokine-dependent effects on all measured parameters could be significantly attenuated using pharmacological- and siRNA-based antioxidant strategies, providing clear evidence that both TNF-α and IL-6 can downregulate HBMvEC barrier phenotype to a comparable degree through activation of the NADPH oxidase pathway.

## Materials and Methods

### Materials

Unless otherwise stated, all reagents were purchased from Sigma-Aldrich (Dublin, IRL). Cytokines (TNF-α, IL-6), apocynin and NSC23766 were purchased from Millipore (Cork, IRL). Primary antisera were purchased from the following sources: Anti-occludin IgG, anti-claudin-5 IgG, and anti-ZO-1 IgG (Bio-Sciences, Dublin, IRL); Anti-VE-cadherin IgG (Abcam, Cambridge, UK); Anti-gp91 IgG, anti-p47 IgG, and anti-GAPDH IgG (Santa Cruz Biotechnology, CA, USA); HRP-conjugated secondary antisera for VE-cadherin, occludin, claudin-5, and GAPDH were purchased from Cell Signalling Technologies Inc. (MA, USA). HRP-conjugated secondary antisera for gp91 and p47 were purchased from Sigma Aldrich. siRNA constructs for gp91 (SC35503, RefSeq NM_000397.3) and p47 (SC29422, RefSeq NM_000265.5) were obtained from Santa Cruz Biotechnology.

### Cell Culture

Culture of primary-derived human brain microvascular endothelial cells (HBMvECs) was based on a modification of the method of Walsh *et al.* [15]. HBMvECs were obtained from Cell Systems Corporation (WA, USA-Cat No. ACBRI 376) and routinely grown in EndoGRO MV Basal Medium (Millipore, Cork, IRL) supplemented with 5% fetal bovine serum, L-Glutamine (10 mM), ascorbic acid (50 µg/ml), heparin sulphate (0.75 U/ml), hydrocortisone hemisuccinate (1 µg/ml), recombinant human epidermal growth factor (5 ng/ml), EndoGRO-LS Supplement (0.2%) and antibiotics (100 µg/ml Mycozap). All cells (passages 5–12) were grown on Attachment Factor-coated tissue culture grade plastic-ware and maintained in a humidified atmosphere of 5% CO_2_/95% air at 37°C.

For experimental purposes, cells were routinely subjected to treatment with either TNF-α or IL-6 at 0–100 ng/ml (0–24 hrs), concentration ranges routinely used in other papers [10], [19]. Post-cytokine treatment, cells were harvested for analysis. For cell lysate preparation, cells were washed thrice in PBS before being scraped into radioimmunoprecipitation assay (RIPA) lysis buffer (64 mM HEPES pH 7.5, 192 mM NaCL, 1.28% w/v Triton X-100, 0.64% w/v sodium deoxycholate, 0.128% w/v sodium dodecyl sulfate, 0.5 M sodium fluoride, 0.5 M EDTA, 0.1 M sodium phosphate, 10 mM sodium orthovanadate, and 1X protease/phosphatase inhibitor cocktail) and transferred into a pre-chilled micro-centrifuge tube. Continuous lysate rotation was applied for 1 hr at 4°C, prior to lysate clarification by centrifugation at 10,000×g for 20 min at 4°C to sediment any triton-insoluble material. Clarified lysates were quantified by BCA assay [20]. All protein lysates were subsequently stored at −80°C pending further analysis.

In conjunction with cytokine experiments, pharmacological agents were employed to dissect the role of both ROS and NADPH oxidase in mediating cytokine-dependent effects on HBMvEC barrier phenotype. These included: 200 U/ml superoxide dismutase (SOD); 200 U/ml catalase (CAT); 1 mM *N*-acetylcysteine (NAC); 10 µM apocynin (APO); and 50 µM NSC23766 (Millipore). Cells were typically pre-treated with these compounds for 1 hr in advance of cytokine treatment, with the compound also remaining on the cells for the duration of the cytokine treatment. Concentrations were selected based on previous usage across the scientific literature [15], [21] in conjunction with statistically minimal effects on cell viability (Figure S1A).

### Flow Cytometry

For analysis of cytokine-dependent ROS generation, confluent HBMvECs were labelled with 5 µM 2′, 7′-dichlorofluorescein diacetate (CFDA) prior to cytokine treatment, or with 3 µM dihydroethidium (DHE) for 30 mins prior to completion of cytokine treatment. Post-treatment, HBMvECs were trypsinized from six-well dishes, pelleted, and washed in FACS buffer (filtered PBS containing 2% fetal bovine serum and 0.1% sodium azide). Cells were then resuspended in 500 µl FACS buffer and read for 10,000 events using a BD FACS Aria. For DHE, excitation and emission wavelengths were 470 nm and 610 nm, respectively (i.e. PE Texas Red spectral range). For CFDA, excitation and emission wavelengths were 492 nm and 517 nm, respectively (i.e. FITC spectral range). All FACS data analysis employed FlowJo software.

For analysis of cell viability under different treatments, an Alexa Fluor 488 Annexin V-Propidium Iodide/Dead Cell Apoptosis Kit (Bio-Sciences) was used according to manufacturer instructions. Briefly, post-treatment HBMvECs were trypsinized and pelleted by centrifugation before being washed in warmed PBS. Cells were re-centrifuged and the pellet resuspended in 100 µl of 1X Annexin-binding buffer. The 100 µl cell suspensions were then transferred to sterile FACS tubes and 5 µl of Alexa Fluor 488 Annexin V plus 1 µl of 100 µg/ml propidium iodide (the latter prepared in 1X Annexin-binding buffer) added. The cells were then incubated for 15 mins at room temperature in the dark. Following incubation, 400 µl of 1X Annexin-binding buffer was added to each tube and the samples were mixed gently. The cells were kept on ice and protected from light until analysed by flow cytometry (FITC and propidium iodide spectral ranges).

### Western Immunoblotting (IB)

Post-cytokine treatment, endothelial cell lysates were harvested, resolved by 10% SDS-PAGE under reducing conditions (8% for ZO-1), and electroblotted as previously described [22]. Membranes were blocked for 60 mins in tris-buffered saline (TBS: 10 mM Tris pH 8.0, 150 mM NaCl) containing 5% w/v bovine serum albumin (BSA) before being incubated overnight in primary antisera with gentle agitation at 4°C. Primary antisera were prepared in TBS (+1% BSA): 0.5 µg/ml anti-occludin, anti-claudin-5, and anti-ZO-1 mouse monoclonal IgGs; 0.2 µg/ml anti-VE-Cadherin mouse monoclonal IgG; 0.4 µg/ml anti-gp91 goat polyclonal IgG; 0.4 µg/ml anti-p47 goat polyclonal IgG; and 0.2 µg/ml anti-GAPDH rabbit monoclonal IgG. Membranes were then washed thrice in TBS containing 0.1% Tween (TBST) before being incubated for 3 hrs in secondary antisera with gentle agitation at room temperature. Secondary antisera were prepared in TBST (+1% BSA): 1∶2000 HRP-conjugated goat anti-mouse IgG (VE-Cadherin, occludin, claudin-5, ZO-1), 1∶2000 HRP-conjugated rabbit anti-goat IgG (gp91, p47), and 1∶3000 HRP-conjugated goat anti-rabbit IgG (GAPDH). Membranes were developed using a Luminata Western HRP kit (Millipore, Cork, IRL) followed by chemiluminescent imaging using a G-Box gel-documentation system (Syngene, UK). Scanning densitometry of Western blots was routinely performed using NIH ImageJ software, with GAPDH routinely employed as a loading control to facilitate densitometric normalization of bands.

### Immunoprecipitation (IP)

Column IP was employed in conjunction with IB to monitor changes in the co-association of NADPH oxidase subunits, gp91 and p47, in response to cytokine treatment of HBMvECs. All IPs were performed using a Co-IP Kit (Pierce, Cheshire, UK) and all relevant beaded agarose columns (i.e. for anti-gp91 and anti-p47 “pull-downs”) were prepared in accordance with manufacturer instructions. Briefly, post-treatment HBMvECs were harvested and lysed, with lysates subsequently diluted down to a final volume of 300 µl using IP Lysis/Wash Buffer. Lysates were then transferred to individual pre-equilibrated columns (containing specific target antisera derivatized to agarose beads), which were subsequently sealed and rotated for 4 hrs at 4°C. Following incubation, the columns were placed in fresh collection tubes and centrifuged at 1000×g for 1 min. Columns were then washed thrice with 200 µl of IP Lysis/Wash Buffer with each wash subjected to an intermittent centrifugation step (1000×g for 1 min). The columns were then transferred to fresh collection tubes and 60 µl of Elution Buffer was added for 5 mins and centrifuged accordingly. The collected eluent was then stored at −80°C for subsequent analysis by IB.

### Transfection

For siRNA transfections (gp91, p47), the Microporator Mini MP-100 system (Life Technologies, UK) was used in accordance with manufacturer instructions. HBMvECs were resuspended into sterile micro-centrifuge tubes at a final concentration of 5×10^5^ cells/100 µl in R buffer and siRNA then added. Following electroporation, the contents of each micro-centrifuge tube was transferred to a designated well of a 6-well dish containing 1 ml of pre-warmed media (−antibiotic) for overnight plating out (final siRNA concentration in each well was 50 nM). After the overnight plating-out period, media was replenished with normal complete media (+antibiotic) and the experimental cytokine treatments typically commenced 36 hrs after transfection. Transfection efficiency in HBMvECs was assessed using an enhanced green fluorescent protein (eGFP) construct in conjunction with flow cytometry and fluorescence microscopy (∼90% efficiency). siRNA knockdown efficiency (0–50 nM range) for both gp91 and p47 was also assessed by Western blotting (Figure S2). Mock transfections were routinely included in all experiments for control purposes.

### Transendothelial Permeability Assay

For analysis of permeability following cytokine treatments, the earlier method of Walsh *et al.* was employed with minor modifications [15]. Briefly, HBMvECs were plated at high density (5×10^5^ cells/well) into Millicell hanging cell culture inserts (Millipore; 6-well format, 0.4 µm pore size, 24 mm filter diameter). At confluency, fresh complete media was added to the upper (abluminal) and lower (subluminal) chambers of the Millicell insert within the 6-well dish (2 ml; upper, 4 ml; lower). Cells within the upper chamber were treated with 0 or 100 ng/ml of either TNF-α or IL-6 for 18 hrs in the absence and presence of pharmacological agents (SOD, CAT, NAC, APO, NSC23766) or siRNA transfection (gp91 or p47). Post-treatment, media in the upper and lower chambers was replenished, fluorescein isothiocyanate (FITC)-labeled 40 kDa dextran was added to the upper chamber (giving a final concentration of 250 µg/ml), and transwell diffusion allowed to proceed. Media samples (28 µl) were collected every 30 mins from the lower subluminal chamber for up to 3 hrs, diluted to a final volume of 400 µl with complete media, and monitored in 96-well format for FITC-dextran fluorescence. A TECAN Safire 2 fluorospectrometer was used with excitation and emission wavelengths set at 490 and 520 nm, respectively. Permeability is presented as % transendothelial exchange of FITC-dextran 40 kDa (%TEE FD40).

### Statistical Analysis

Results are expressed as mean±s.d. Experimental points were typically performed in triplicate with a minimum of three independent experiments (n = 3). Statistical comparisons between control and experimental groups was by ANOVA in conjunction with a Dunnett’s *post-hoc* test for multiple comparisons. A Student’s *t*-test was also routinely employed for pairwise comparisons. A value of *P*≤0.05 was considered significant.

## Results

### TNF-α and IL-6 reduce expression of VE-cadherin, occludin and claudin-5 in a dose-dependent manner in HBMvECs

**Figure 1:**
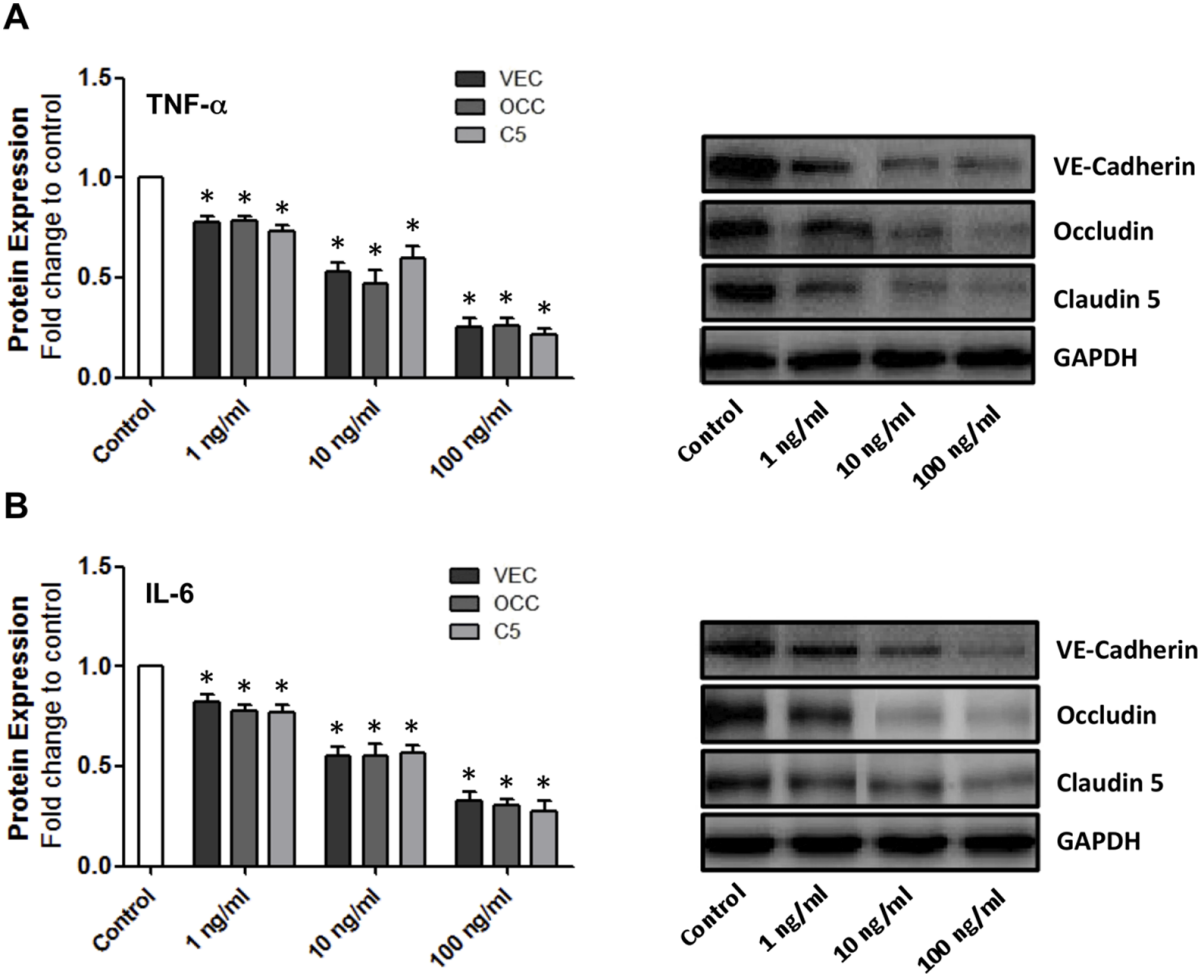
Dose-dependent effect of cytokines on interendothelial junction protein expression in HBMvECs. Confluent cells were treated with TNF-α (**A**) or IL-6 (**B**) (0–100 ng/ml, 18 hrs). Post-treatment, whole cell protein lysates were harvested for Western blotting. Histograms represent the densitometric fold change in relative protein expression for VE-cadherin, occludin and claudin-5 (bars reading left to right) in response to increasing concentration of cytokine. *P≤0.05 versus untreated control. All gels are representative.

The effect of proinflammatory cytokines on the expression of interendothelial junction proteins was initially monitored. Treatment of confluent HBMvECs with 0–100 ng/ml of either TNF-α (Figure 1A) or IL-6 (Figure 1B) for 18 hrs demonstrated a dose-dependent reduction in expression of the interendothelial complex proteins VE-cadherin, occludin and claudin-5, as monitored by Western blotting. At the upper treatment concentration of 100 ng/ml, either cytokine caused a maximal reduction in protein expression level of approximately 75% for each junctional protein. Finally, it can be noted that all of the above trends were also observed following 6 hrs cytokine treatment (Figure S3).

### TNF-α and IL-6 induce ROS generation in both a time- and dose-dependent manner in HBMvECs

**Figure 2:**
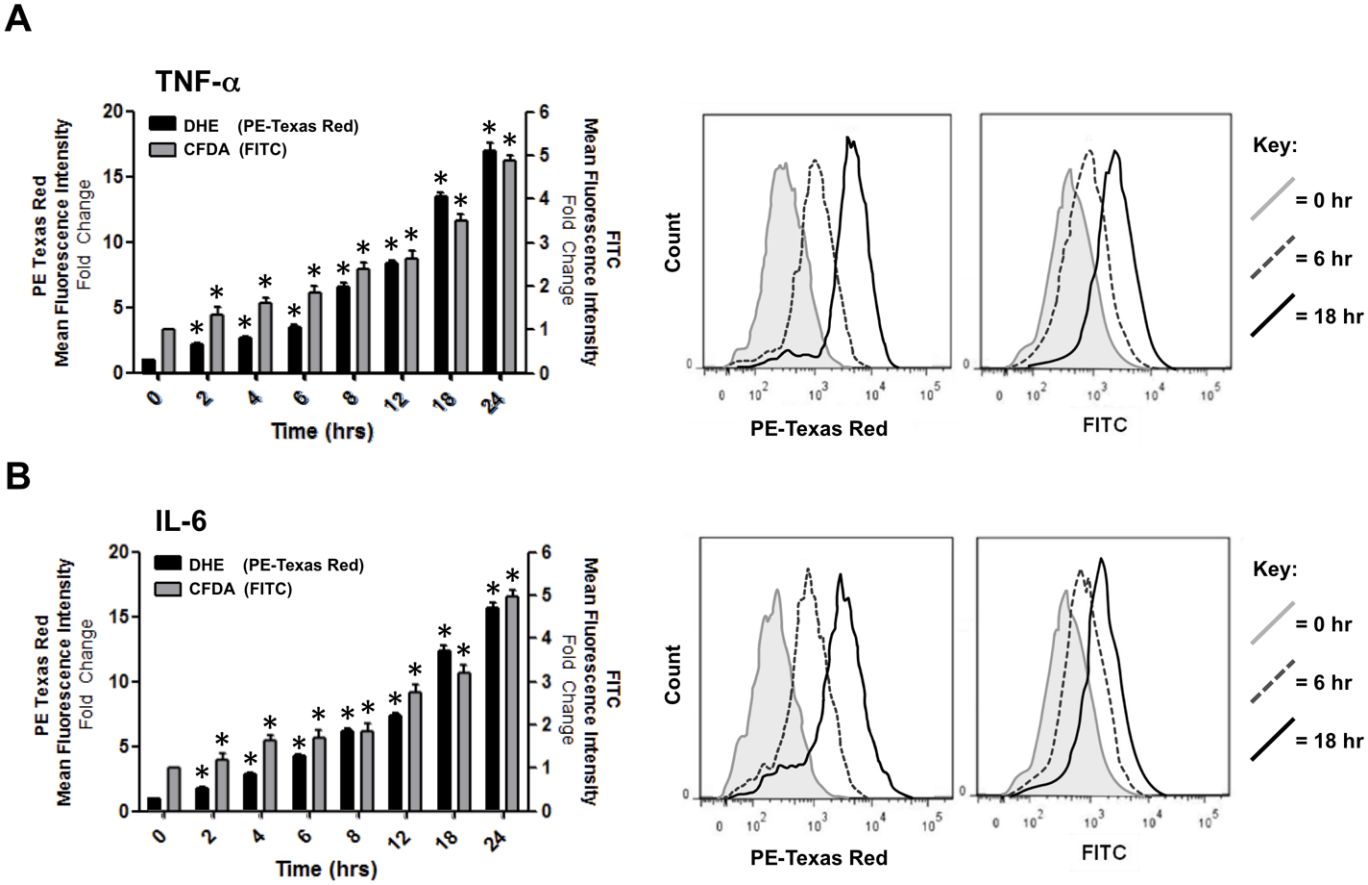
Time-dependent effect of cytokines on ROS generation in HBMvECs. Confluent cells were treated with TNF-α (**A**) or IL-6 (**B**) (100 ng/ml, 0–24 hrs) and ROS generation monitored by flow cytometry using fluorescent ROS-detecting compounds, DHE (PE Texas Red detector) or CFDA (FITC detector). Histograms (LHS – left hand side) represent the fold change in fluorescent signal normalized to 0 hr DHE. Representative FACS scans (RHS – right hand side) are shown for 0, 6, and 18 hr timepoints only. Grey shaded scan indicates 0 hr control (full key to scans on RHS). *P≤0.05 versus 0 hr DHE.

**Figure 3:**
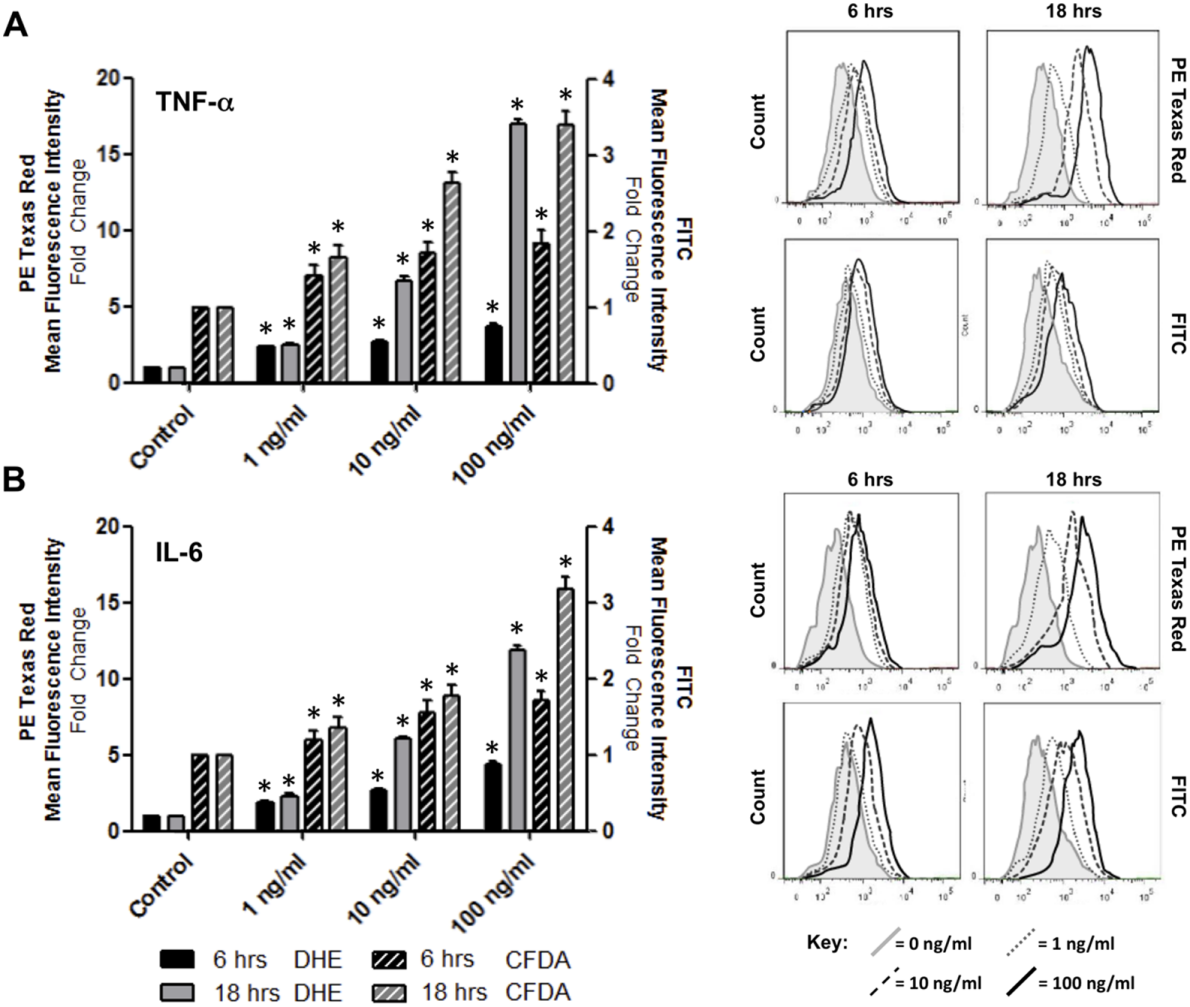
Dose-dependent effect of cytokines on ROS generation in HBMvECs. Confluent cells were treated with TNF-α (**A**) or IL-6 (**B**) (0–100 ng/ml, 6 or 18 hrs) and ROS generation monitored by flow cytometry using fluorescent ROS-detecting compounds, DHE (PE Texas Red detector) or CFDA (FITC detector). Histograms (LHS) represent the fold change in fluorescent signal normalized to untreated DHE control at 6 or 18 hrs. Representative FACS scans (RHS) are shown for both 6 and 18 hr treatments with increasing cytokine concentration evident from left to right. Grey shaded scan indicates untreated control (full key beneath scans). *P≤0.05 versus untreated DHE 6 or 18 hr controls.

The effect of proinflammatory cytokines on ROS generation was next monitored. Treatment of confluent HBMvECs with 100 ng/ml of either TNF-α (Figure 2A) or IL-6 (Figure 2B) for 0–24 hrs demonstrated a similar time-dependent fold increase in intracellular ROS levels, as monitored by flow cytometry using both DHE (PE Texas Red) and CFDA (FITC) fluorescent detectors. For experimental consistency, all subsequent experiments were conducted under both a short (6 hrs) and long (18 hrs) cytokine exposure time (and unless otherwise stated, at 100 ng/ml). Figure S1B demonstrates the negligible impact on cell viability following cytokine treatment for these timepoints (although it should be noted that there were also negligible effects on cell viability after 24 hrs treatment with either cytokine at 100 ng/ml). The dose-dependent nature of ROS generation in HBMvECs by either TNF-α (Figure 3A) or IL-6 (Figure 3B) was also clearly evident over a 0–100 ng/ml cytokine dose range (monitored at both 6 and 18 hr time-points, and again using both CFDA and DHE fluorescent detectors).

### Cytokine-dependent ROS generation downregulates expression of interendothelial junction proteins in HBMvECs

**Figure 4:**
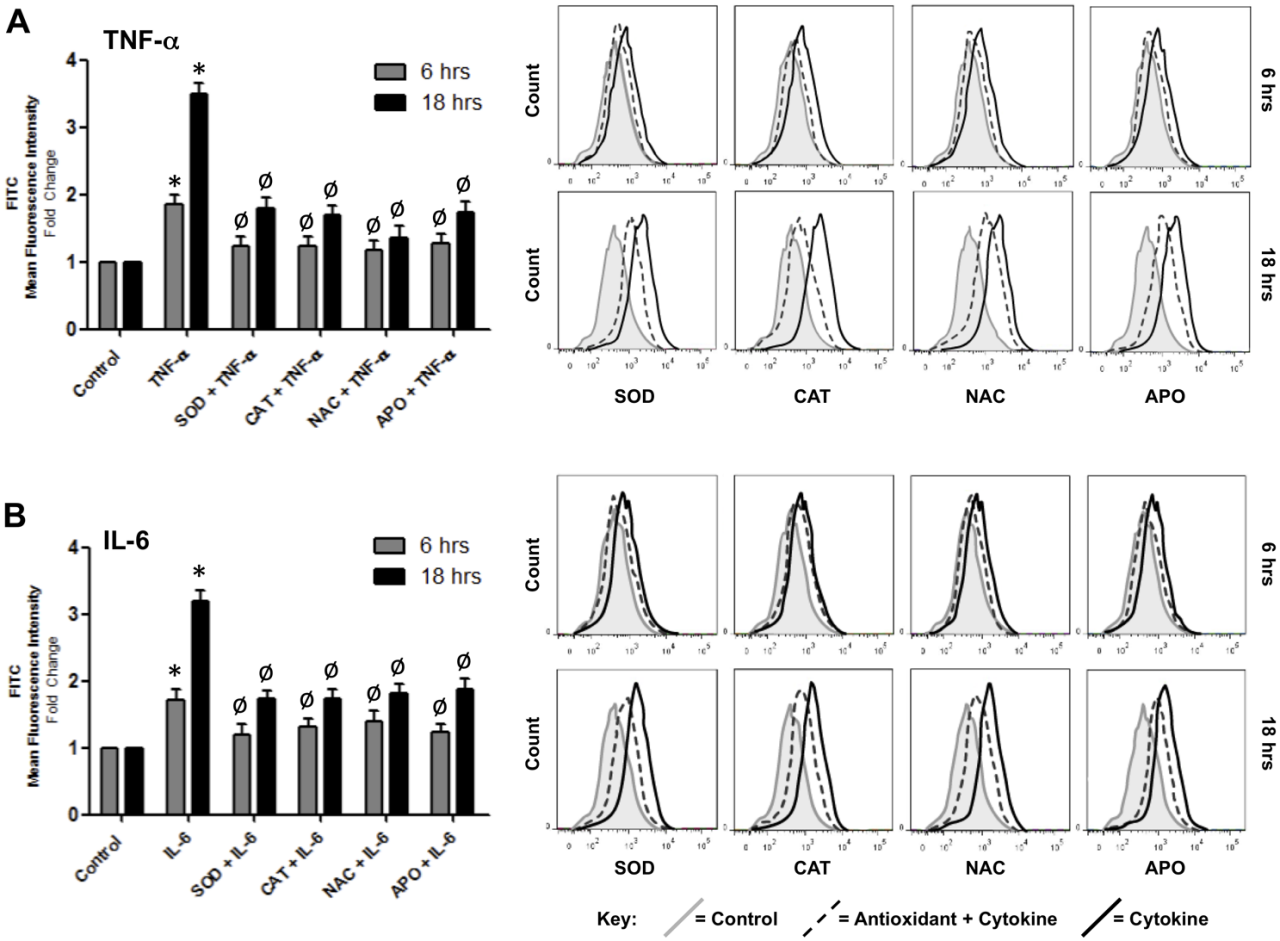
Effect of ROS depleting agents on cytokine-induced ROS generation in HBMvECs. Confluent cells were pre-treated with either SOD (200 U/ml), CAT (200 U/ml), NAC (1 mM) or APO (10 µM), followed by treatment with TNF-α (**A**) or IL-6 (**B**) (100 ng/ml, 6 or 18 hrs). ROS production was subsequently monitored by flow cytometry using ROS-detecting CFDA. Histograms (LHS) represent the fold change in fluorescent signal normalized to untreated control at 6 or 18 hrs. Representative FACS scans (RHS) are shown for both 6 and 18 hr treatments. Grey shaded scan indicates untreated control (full key beneath scans). *P≤0.05 versus untreated 6 or 18 hr controls. ^Ø^ *P*≤0.05 versus cytokine without ROS depleting agent.

**Figure 5:**
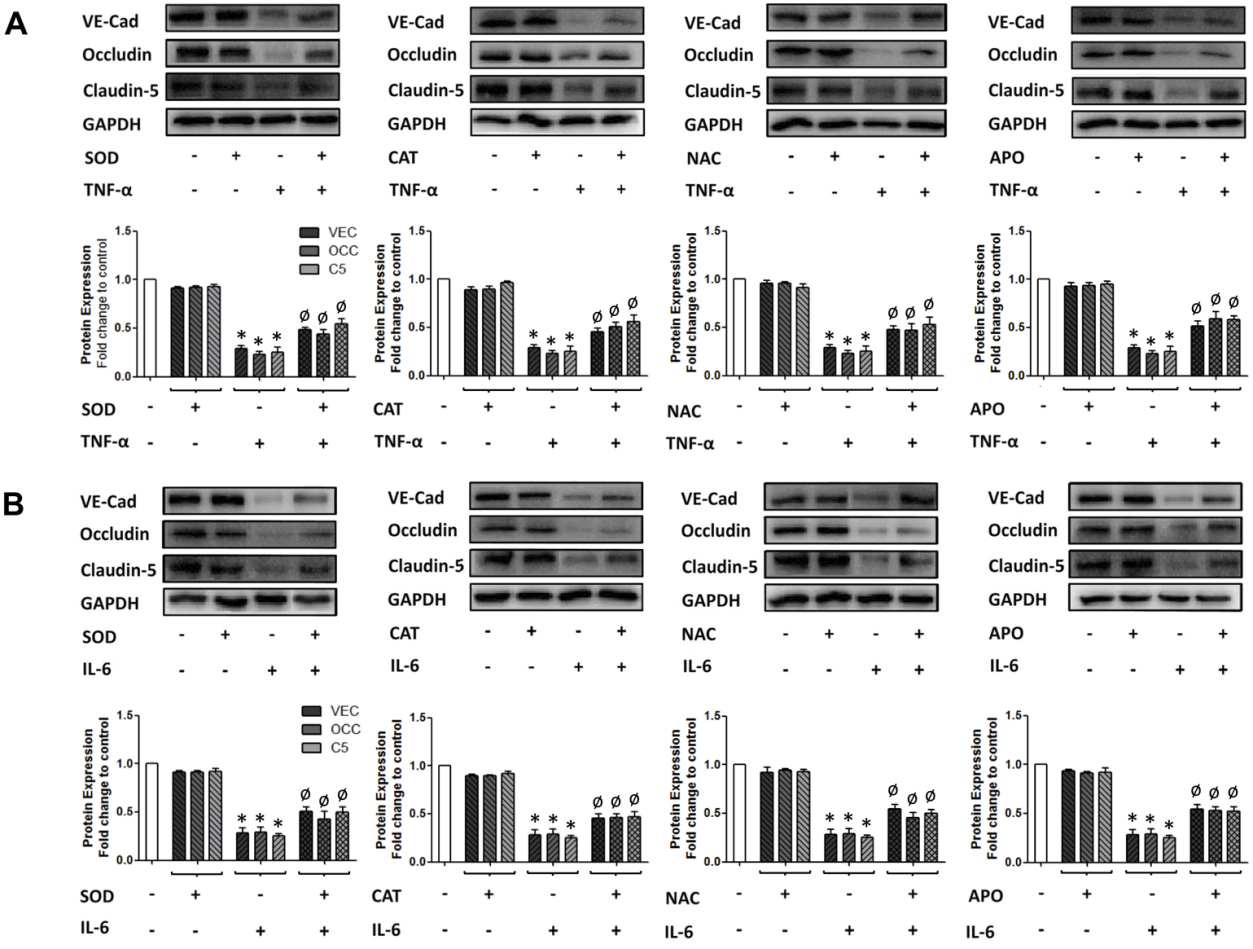
Effect of ROS depleting agents on cytokine-induced downregulation of interendothelial junction protein expression in HBMvECs. Confluent cells were pre-treated with either SOD (200 U/ml), CAT (200 U/ml), NAC (1 mM) or APO (10 µM), followed by treatment with TNF-α (**A**) or IL-6 (**B**) (100 ng/ml, 18 hrs). Post-treatment, whole cell protein lysates were harvested for Western blotting. Histograms below gels represent the densitometric fold change in relative protein expression for VE-cadherin, occludin and claudin-5 (bars reading left to right) in response to cytokine treatment in the absence and presence of ROS depleting agent. *P≤0.05 versus untreated control. ^Ø^ *P*≤0.05 versus cytokine without ROS-depleting agent. All gels are representative.

The relationship between parallel cytokine-dependent events, namely the induction of ROS generation and the downregulation of interendothelial junction protein expression, was next investigated using a range of ROS depleting pharmacological agents. Confluent HBMvECs were pre-treated with either SOD, CAT, NAC or APO before being treated with 100 ng/ml of either TNF-α or IL-6 for up to 18 hrs, after which cells were harvested and monitored for ROS production by flow cytometry (necessitating cell pre-labelling with ROS-sensitive CFDA) or for protein expression analysis by Western blotting. Pre-treatment with ROS depleting agents maximally attenuated the ROS producing actions of TNF-α (Figure 4A) and IL-6 (Figure 4B) by 88% and 65%, respectively. It can be noted that similar trends were also observed utilising DHE as the ROS-sensitive fluorescent label (Figure S4). Treatment for 18 hrs with 100 ng/ml of either cytokine led to a significant reduction (up to 75%) in the expression of the interendothelial complex proteins VE-cadherin, occludin and claudin-5 (Figure 5). Moreover, pre-treatment of cells with ROS depleting agents consistently recovered the cytokine-mediated downregulation of these junctional proteins by approximately 44% for both TNF-α (Figure 5A) and IL-6 (Figure 5B), trends that were also observed following 6 hrs cytokine treatment (data not shown).

### TNF-α and IL-6 upregulate NADPH oxidase activation in HBMvECs

**Figure 6:**
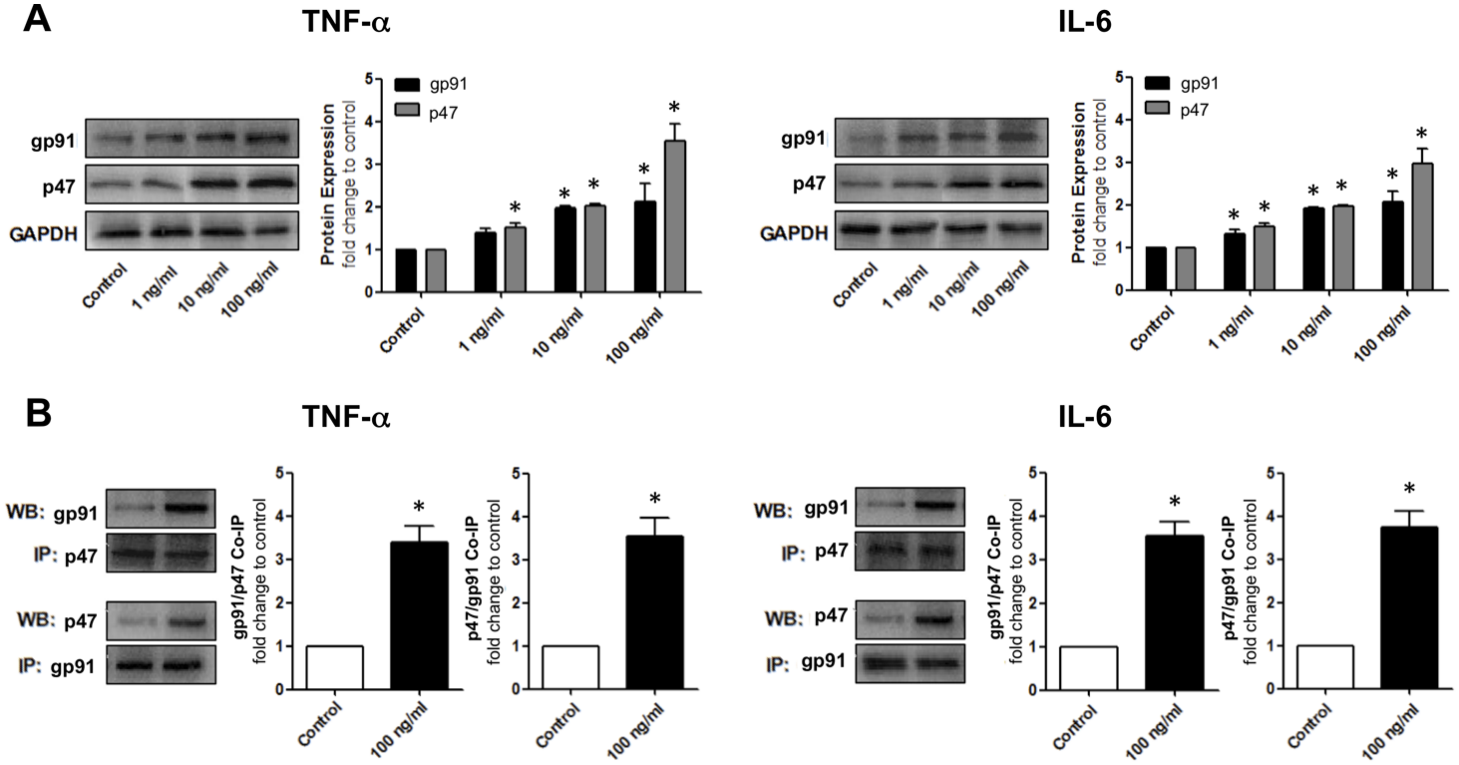
Effect of cytokines on NADPH oxidase activation in HBMvECs. (**A**) Confluent cells were treated with TNF-α (LHS) or IL-6 (RHS) (0–100 ng/ml, 18 hrs) prior to harvesting of whole cell protein lysates for Western blotting. Histograms represent the densitometric fold change in relative protein expression for gp91 and p47 in response to increasing concentrations of cytokine. (**B**) Confluent cells were also treated with TNF-α (LHS) or IL-6 (RHS) (100 ng/ml, 18 hrs) prior to harvesting of whole cell protein lysates for co-IP. Histograms represent the densitometric fold change in gp91/p47 co-association in response to cytokine treatment. For each cytokine; LHS histogram = IP p47, WB gp91, RHS histogram = IP gp91, WB p47. *P≤0.05 versus untreated control. All gels are representative.

The effect of proinflammatory cytokines on the expression and co-association of NADPH oxidase subunits, gp91 and p47, was next investigated. These subunits consititute membrane-bound and cytosolic components of the NADPH oxidase complex, respectively. Their coassembly with other subunits (e.g. p22 and p67) into a functional NADPH oxidase complex are essential to enable the heme group coordination needed for mediating electron transfer to molecular oxygen to generate superoxide (O_2_^−^) [23]. As such, their expression and co-association are a useful index of NADPH oxidase activation. Following treatment of confluent HBMvECs with 0–100 ng/ml of either TNF-α or IL-6 for 18 hrs, cells were harvested for protein expression analysis by Western blotting. We observed a dose-dependent increase in protein expression for both gp91 (up to 2.1-fold for both cytokines at 100 ng/ml) and p47 (up to 3.5-fold and 3.0-fold at 100 ng/ml of TNF-α and IL-6, respectively) (Figure 6A). In further experiments, cells were treated with 100 ng/ml of either TNF-α or IL-6 for 18 hrs before being harvested for analysis of gp91/p47 co-association by co-IP. When either protein was employed as the ‘pull-down’ target, we observed significantly elevated co-association of both subunits in response to both TNF-α (up to 3.5-fold) and IL-6 (up to 3.8-fold) treatment (Figure 6B). Finally, it can be noted that all of the above trends were also observed following 6 hrs cytokine treatment (Figure S5).

### Cytokine-dependent NADPH oxidase activation downregulates expression of interendothelial junction proteins in HBMvECs

**Figure 7:**
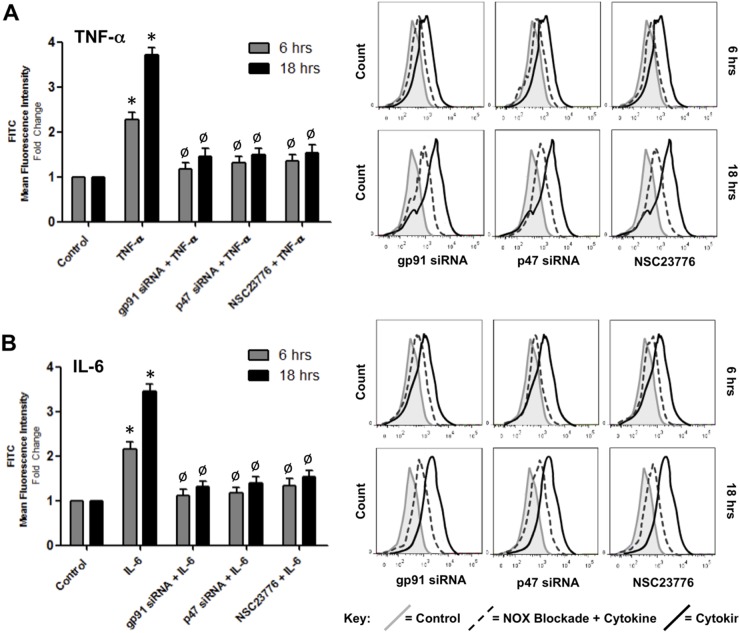
Effect of NADPH oxidase blockade on cytokine-induced ROS generation in HBMvECs. Confluent cells were either transfected with siRNA targeting gp91 or p47, or were pre-treated with NSC23776 (50 µM) for 1 hr prior to stimulation with TNF-α (**A**) or IL-6 (**B**) (100 ng/ml, 6 or 18 hrs). ROS generation was subsequently monitored by flow cytometry using ROS-detecting CFDA. Histograms (LHS) represent the fold change in fluorescent signal normalised to untreated control at 6 or 18 hrs. Representative FACS scans (RHS) are shown for both 6 and 18 hr treatments. Grey shaded scan indicates untreated control (full key beneath scans). *P≤0.05 versus untreated control at 6 or 18 hrs. ^Ø^ *P*≤0.05 versus cytokine without NADPH oxidase blockade.

**Figure 8:**
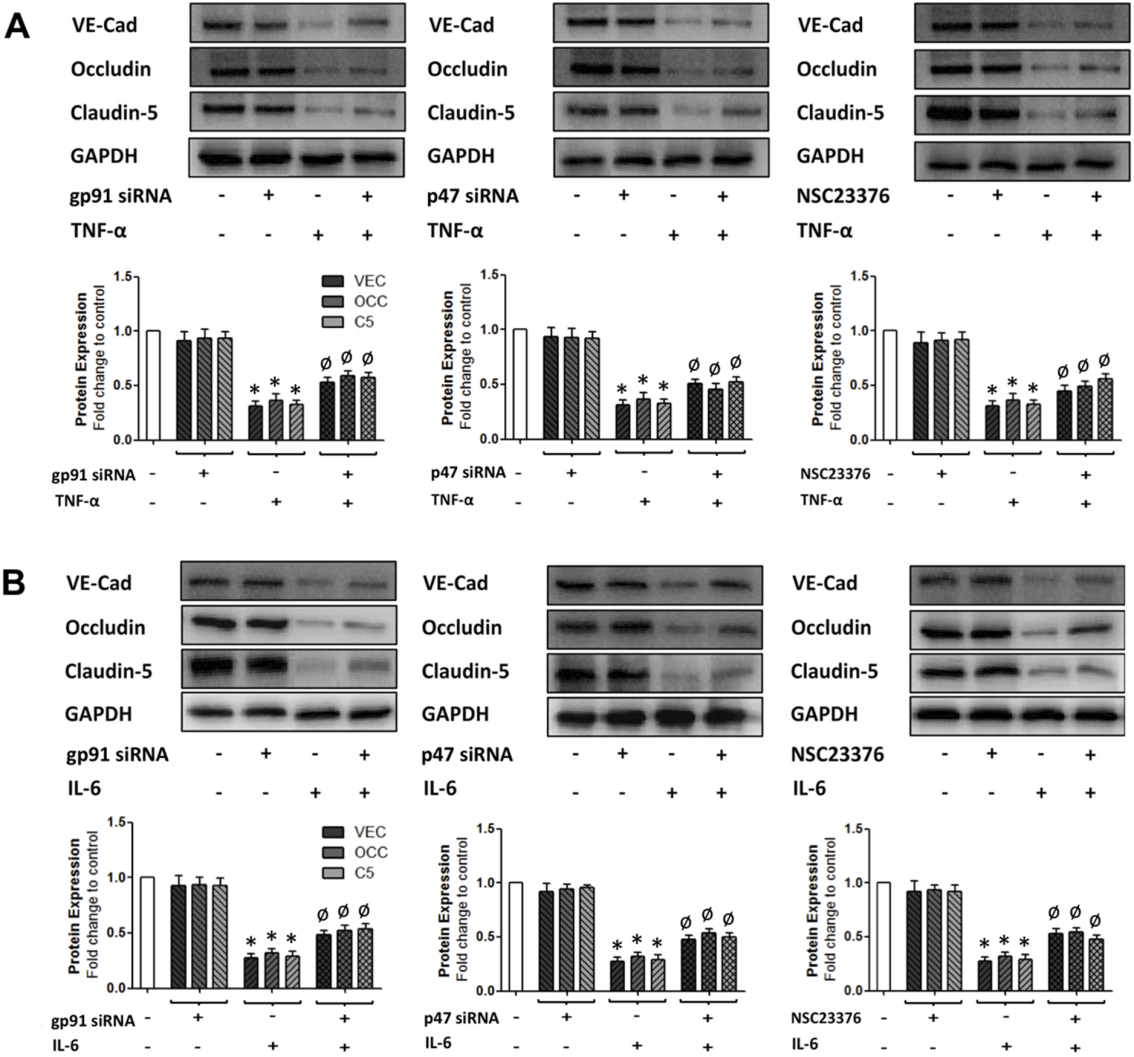
Effect of NADPH oxidase blockade on cytokine-induced downregulation of interendothelial junction protein expression in HBMvECs. Confluent cells were either transfected with siRNA targeting gp91 or p47, or were pre-treated with NSC23776 (50 µM) followed by treatment with TNF-α (**A**) or IL-6 (**B**) (100 ng/ml, 18 hrs). Post-treatment, whole cell protein lysates were harvested for Western blotting. Histograms below gels represent the densitometric fold change in protein expression for VE-cadherin, occludin and claudin-5 in response to NADPH oxidase blockade. *P≤0.05 versus untreated control. ^Ø^ *P*≤0.05 versus cytokine without NADPH oxidase blockade. All gels are representative.

In view of the fact that cytokine treatment enhances the expression and co-association of the NADPH oxidase subunits, gp91 and p47, we next decided to investigate the effect of selectively ablating the expression of these subunits on the ability of both TNF-α and IL-6 to downregulate junctional protein expression. Custom siRNA constructs directed towards gp91 and p47 were initially pre-tested in cultured HBMvECs and each demonstrated up to 80% knockdown of subunit protein expression at 50 nM, as monitored by Western blotting (Figure S2). HBMvECs transfected with either gp91 or p47 siRNA (followed by cell pre-labelling with ROS-sensitive CFDA) demonstrated significantly attenuated ROS generation (>75%) in response to treatment with 100 ng/ml of either TNF-α (Figure 7A) or IL-6 (Figure 7B) for 6 or 18 hrs, as monitored by flow cytometry. It can be noted that similar trends were also observed utilising DHE as the ROS-detecting label (Figure S6). NSC23766-mediated blockade of Rac1 activation, a feature of NADPH oxidase subunit recruitment to the plasma membrane, had an identical effect to either gp91 or p47 knockdown (Figure 7). Following transfection and cytokine treatments, cells were also harvested for protein expression analysis by Western blotting. Treatment for 18 hrs with 100 ng/ml of either cytokine led to a significant reduction (up to 75%) in the expression of interendothelial VE-cadherin, occludin and caudin-5 (Figure 8). Moreover, siRNA knockdown of gp91 or p47, or blockade of Rac1 activation, consistently recovered the cytokine-mediated downregulation of these junctional proteins by approximately 40% for both TNF-α (Figure 8A) and IL-6 (Figure 8B), trends that were also observed following 6 hrs cytokine treatment (data not shown).

### TNF-α and IL-6 increase HBMvEC monolayer permeability in a dose-dependent manner via NADPH oxidase activation

**Figure 9:**
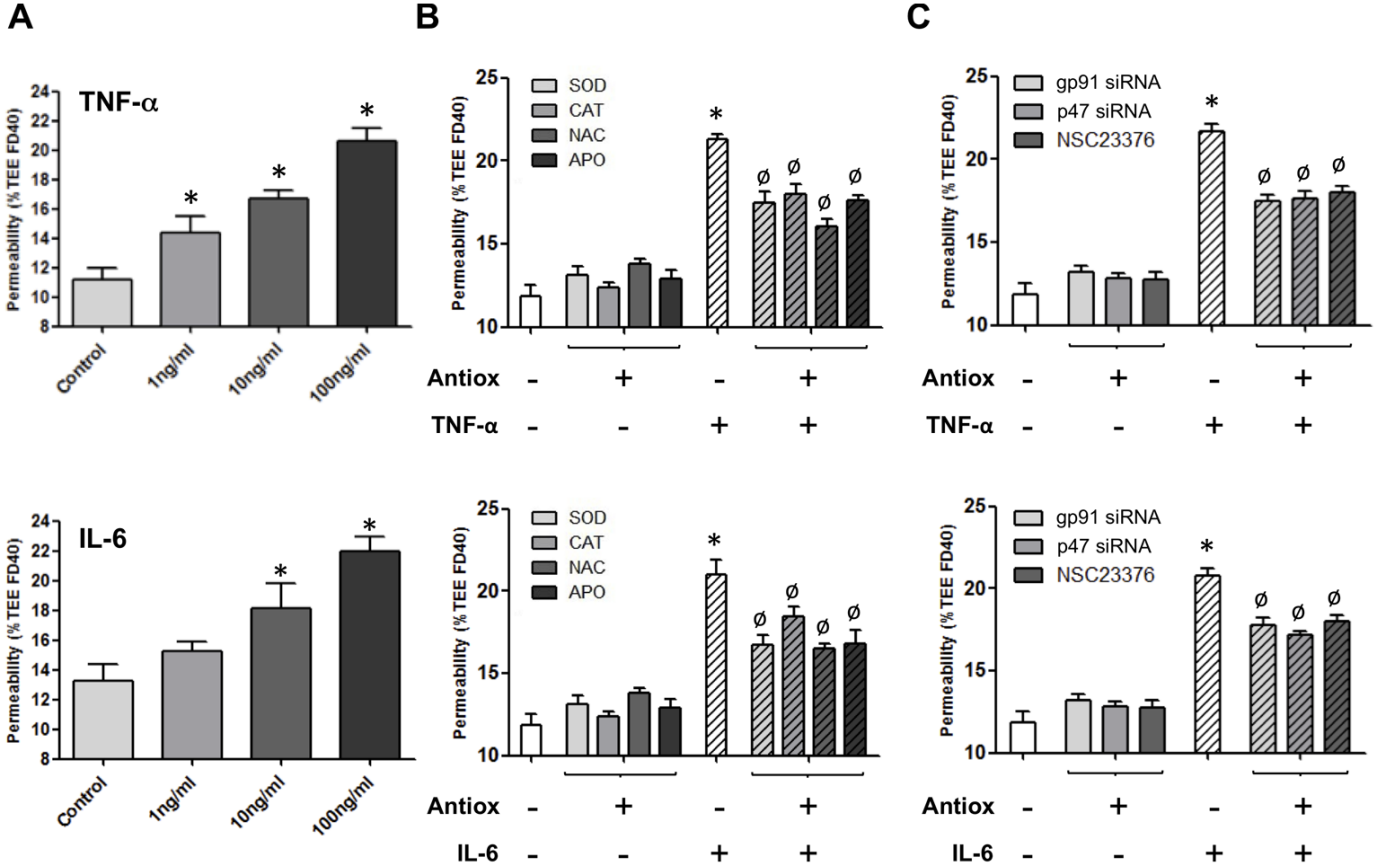
Effect of antioxidant strategies on cytokine-induced HBMvEC barrier disruption. (**A**) Confluent cells were treated with TNF-α (top) or IL-6 (bottom) (0–100 ng/ml, 18 hrs). Post-treatment, HBMvEC monolayer permeability was monitored by transendothelial permeability assay. Histograms represent the increase in % Transendothelial Exchange of FITC-Dextran 40 kDa (%TEE FD40) in response to increasing concentration of cytokine. (**B, C**) Prior to treatment with TNF-α (top) or IL-6 (bottom) (100 ng/ml, 18 hrs), confluent cells were pre-treated with either; (**B**) SOD (200 U/ml), CAT (200 U/ml), NAC (1 mM) or APO (10 µM); or (**C**) gp91 siRNA, p47 siRNA, or NSC23766 (50 mM). Following cytokine treatment, HBMvEC permeability was monitored. Histograms represent the change in permeability (%TEE FD40) in response to cytokines in the absence and presence of antioxidant treatment. *P≤0.05 versus untreated controls. ^Ø^ *P*≤0.05 versus cytokine without antioxidant treatment.

The effect of proinflammatory cytokines on HBMvEC monolayer permeability was next investigated. Treatment of confluent HBMvECs with 0–100 ng/ml of either TNF-α or IL-6 for 18 hrs demonstrated a dose-dependent increase in endothelial permeability, as monitored by transendothelial permeability assay with FITC-dextran 40 kDa (Figure 9A). In a final series of experiments, the effect of antioxidant strategies on the endothelial permeabilizing effects of both cytokines was investigated. Pre-treatment of confluent HBMvECs with ROS depleting agents (SOD, CAT, NAC or APO) maximally attenuated the permeabilizing effects of TNF-α and IL-6 (100 ng/ml, 18 hrs) by 50% and 45%, respectively (Figure 9B). Similarly, siRNA knockdown of gp91 or p47, or blockade of Rac1 activation, maximally attenuated the permeabilizing effects of TNF-α and IL-6 by 47% and 53%, respectively (Figure 9C). Finally, it can be noted that all of the above trends were also observed following 6 hrs cytokine treatment (Figure S7).

## Discussion

Much evidence points to the involvement of proinflammatory cytokines in the pathogenesis of neurological disorders [5], [24], although their precise contribution to the BBB disruption that invariably accompanies such disorders is still unclear. Whilst some studies indicate that cytokine-induced endothelial permeabilization may involve alteration in the expression and/or distribution of interendothelial junction proteins, there are several gaps and inconsistencies in relation to this subject within the existing BBB literature: (i) many reported observations are non-quantitative in nature; (ii) considerable variation exists across different models – from peripheral to cerebrovascular endothelia – with respect to effects on protein expression and paracellular permeability following cytokine treatments; (iii) there is a noticeable scarcity of cytokine dose- and time-dependency studies, as well as cytokine cross-comparative studies, in relevant BBB models; (iv) most observations have been based on non-human BBB models; (v) there has been an arguably disproportionate focus in the literature on the proinflammatory effects of TNF-α on BBB dynamics, with considerably lesser focus on other relevant cytokines such as IL-6; and (vi) many studies lack mechanistic clarity. To address these shortcomings, the present study employed primary-derived HBMvECs to comprehensively compare/contrast the time- and dose-dependent effects of both TNF-α and IL-6 on the expression of the interendothelial junction proteins VE-cadherin, occludin, and claudin-5, in parallel with their effects on HBMvEC permeability. As neurological disorders frequently manifest elevated ROS generation (a known upstream event in cytokine signaling within brain-derived microvascular endothelial cells [25]), the putative coupling of NADPH oxidase-dependent ROS generation to the cytokine-induced modulation of HBMvEC barrier phenotype was also investigated.

Prior to experimentation, the expression of receptors for both TNF-α (TNF-R1 and TNF-R2) and IL-6 (gp130) was confirmed in our cultured HBMvECs (data not shown). Moreover, numerous studies have documented the responsiveness of cultured vascular endothelial cells to exogenous treatment with either cytokine [7], [10], [19], [26], [27]. Treatment of confluent HBMvECs with either cytokine consistently demonstrated a significant dose-dependent reduction in the expression of VE-cadherin, occludin and claudin-5 at the level of both protein (up to 75% at 100 ng/ml cytokine) and mRNA (data not shown), in parallel with a dose-dependent increase in HBMvEC permeability. TNF-α and IL-6 were also seen to decrease the expression of TJ-associated zonula occludens 1 (ZO-1) in a dose-dependent manner (Figure S8). These results confirm that both TNF-α and IL-6 can downregulate human brain microvascular endothelial barrier function *in vitro* in a dose-dependent manner through modulation of paracellular pathway-associated AJ (VE-cadherin) and TJ (occludin, claudin-5, ZO-1) protein complexes at the transcriptional and translational levels. In agreement with these findings, recent studies have demonstrated the ability of TNF-α to decrease the expression of TJ proteins in mouse brain endothelial cells [28] and immortalized human hCMEC/D3 cells [29], [30], whilst both cytokines have also been shown to increase the permeability of cultured endothelial cells [7], [19]. Similarly, Cohen *et al.* have demonstrated the ability of IL-6 to decrease occludin and claudin-5 expression in ovine cerebral microvessels *ex vivo* [31], whilst a role for TNF-α in BBB permeabilization in an *in vivo* mouse model has recently been reported by Wilson *et al.* [32]. In contrast to our findings however, the aforementioned study by Cohen *et al.* demonstrated that IL-6 concentrations below 100 ng/ml did not reduce protein expression, whilst 10 ng/ml of IL-6 was actually seen to increase claudin-5 expression in cerebral microvessels from yearling sheep [31]. In other contrasting studies, a lack of effect on VE-cadherin expression has been reported for hCMEC/D3 cells treated with similar concentrations of TNF-α [30], whilst a recent study by Aveleira *et al.* demonstrates significant upregulation of occludin protein expression in bovine retinal microvascular endothelial cells following TNF-α treatment [11]. In a related study, albeit using human umbilical vein endothelial cells (HUVECs), TNF-α treatment for 24 hrs significantly decreased occludin protein expression, but not that of claudin-5 or ZO-1, although the cell-cell border localization of all three proteins was severely disrupted [12]. Interestingly, maximal TNF-α -induced permeabilization of HAECs in this latter study was achieved at just 10 ng/ml cytokine (as opposed to 100 ng/ml for our HBMvECs). Intrinsic differences between study models (e.g. human versus non human, micro- versus macrovascular etc) presumably accounts for these contrasting observations. Moreover, findings from *ex vivo* and *in vivo* models, being highly sensitive to potential confounders, should be interpreted with caution when assessing the effects of proinflammatory cytokines on the BBB. For example, TNF-α can act on all cells within the neurovascular unit (astrocytes, pericytes, neurons and microvascular endothelial cells) to elicit cellular remodeling, nitric oxide- and glutamate-mediated neurotoxicity, and endothelin-1 upregulation, all of which can contribute directly/indirectly to cerebrovascular endothelial barrier dysfunction [33]–[36], whilst several studies also report evidence of a *neuroprotective* role for TNF-α in the brain for review see [37].

**Figure 10:**
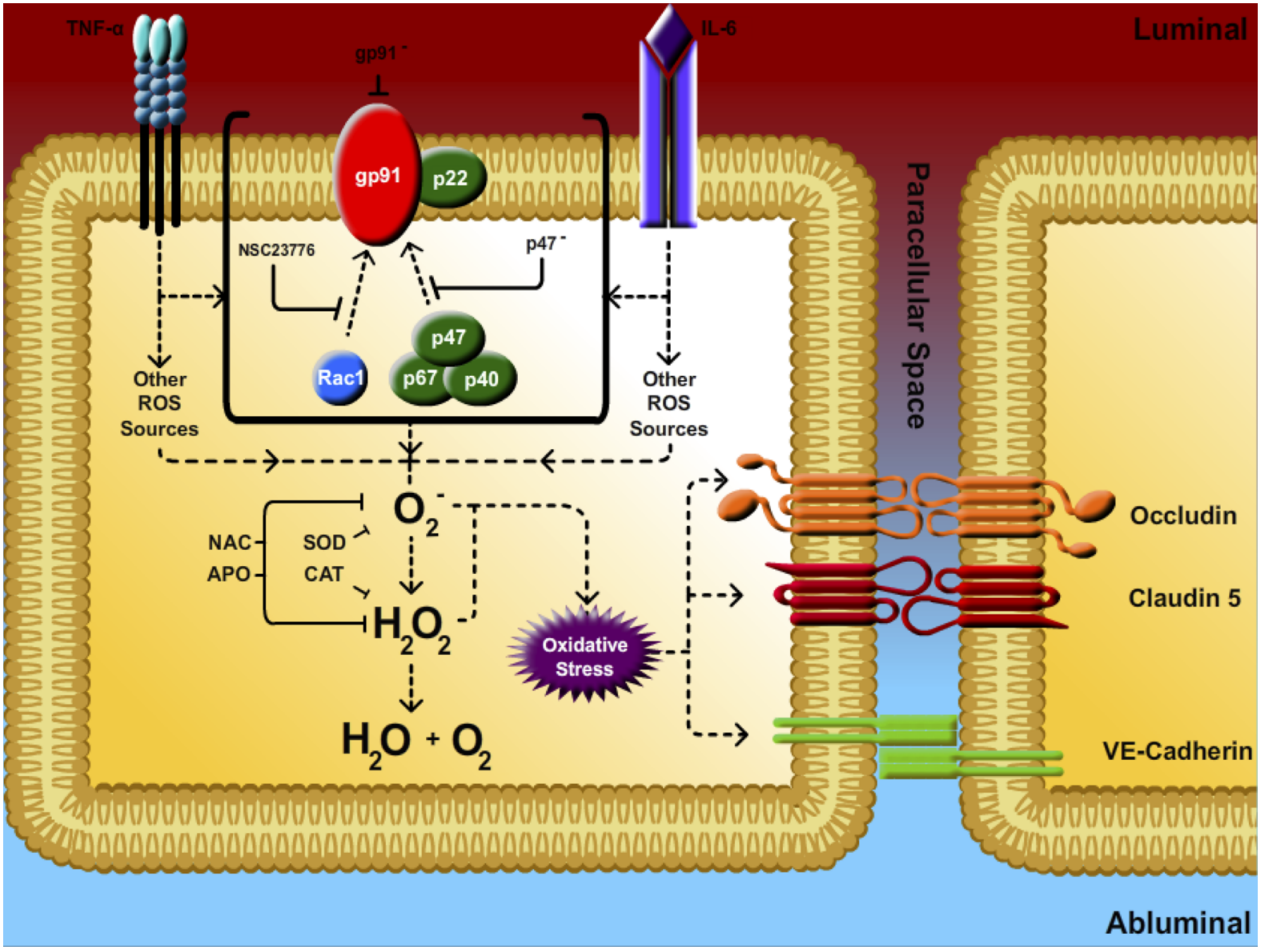
Antioxidant strategies. Summary of antioxidant strategies employed to attenuate the endothelial permeabilizing effects of proinflammatory cytokines. Key: ROS, reactive oxygen species; O_2_^−^, superoxide; H_2_O_2_, hydrogen peroxide; SOD, superoxide dismutase; CAT, catalase; NAC, *N*-acetylcysteine; APO, apocynin; NSC23766, Rac-1 inhibitor; gp91^−^, gp91 siRNA; p47^−^, p47 siRNA.

In order to clarify the mechanism underlying the BBB weakening actions of TNF-α and IL-6, the putative contribution of ROS signalling was next considered using a range of antioxidant strategies (summarized in Figure 10). By employing flow cytometry in conjunction with either of two distinct ROS-detecting agents (DHE and CFDA), our studies clearly demonstrate a similar dose- and time-dependent pattern of ROS generation for both cytokines in HBMvECs, events which could be strongly attenuated using a range of ROS depleting pharmacological agents (SOD, CAT, NAC, APO). In subsequent studies, the same ROS depleting agents were found to attenuate to a comparable extent the converse decrease in AJ/TJ protein levels and increase in HBMvEC permeability following either TNF-α or IL-6 treatment. Based on these observations, we conclude that the reduced expression and barrier function in HBMvECs is functionally coupled in-part to the cytokine-mediated generation of ROS (i.e. superoxide). Within the vasculature, the contribution of ROS to normal physiological signaling processes and gene expression, as well as to proinflammatory phenotype and pathology, is well established [38], [39]. Numerous published studies demonstrating ROS generation by TNF-α in brain microvascular endothelial cells concur with our observations [40], [41], whilst a limited number of studies highlight the ability of ROS depleting agents such as NAC and SOD to attenuate the endothelial permeabilizing actions of this proinflammatory cytokine [16], [42]. The ROS-inducing abilities of IL-6 within the endothelium however, are less well understood. An earlier study by Wassmann *et al.* demonstrated that IL-6 could enhance AT_1_R gene expression and angiotensin-II-mediated induction of ROS both in cultured vascular smooth muscle cells and in a C57BL/6J mouse model [43]. To our knowledge however, the present study is the first to comprehensively profile time- and dose-dependent ROS generation in HBMvECs by IL-6 and to link this to the associated downregulation of BBB phenotype.

In response to a variety of pathophysiological stimuli (including cytokines), activation of NADPH oxidase leading to oxidant signaling is now well recognized in vascular endothelial cells [17]. In a final series of experiments, we therefore sought to confirm a role for NADPH oxidase activation in the HBMvEC barrier dysfunction observed following treatment with either cytokine. Our data demonstrated that treatment of HBMvECs with either TNF-α or IL-6 significantly increased the expression and co-association of gp91 and p47, pivotal subunits within the NADPH oxidase complex. This is consistent with an earlier study by Gertzberg *et al.* demonstrating increased expression and co-localization of p22 and p47 in bovine lung microvascular endothelial cells in response to TNF-α treatment [16]. Likewise, TNF-α-dependent increases in both the co-association of p47 with gp91, as well as in gp91 expression, have also been reported, albeit in endothelial cells of pulmonary artery origin [18], [44]. Interestingly, whilst numerous studies have linked NADPH oxidase-dependent ROS generation to the elevated expression and release of IL-6 in endothelial cells [45], [46], to our knowledge there are no previous studies clearly documenting IL-6-mediated activation of NADPH oxidase. It can be noted however that functional coupling between NADPH oxidase and the Janus Kinase/Signal Transducer and Activator of Transcription (JAK/STAT) pathway through which IL-6 is known to operate [47] has been established in vascular cells [48], [49] and, given the proinflammatory nature of IL-6, points to the likelihood of bidirectional regulation between IL-6 signaling and NADPH oxidase-dependent ROS generation. In subsequent studies, targeted blockade of NADPH oxidase using either siRNA (gp91, p47) or pharmacological (Rac1) strategies was seen to attenuate to a comparable extent the converse decrease in AJ/TJ protein levels and increase in HBMvEC permeability following either TNF-α or IL-6 treatment. Finally, the quantitatively similar effects obtained using either pharmacological ROS depleting agents or siRNA targeted NADPH oxidase blockade leads us to conclude that NADPH oxidase, as opposed to other ROS-generating systems (e.g. xanthine oxidase), are involved in the HBMvEC barrier dysfunction arising from proinflammatory cytokine treatment.

In summary, a comprehensive investigation comparing the permeabilizing actions of TNF-α and IL-6 in human brain microvascular endothelial cells is presented, with novel findings confirming how *either* cytokine can similarly downregulate the expression of interendothelial adherens and tight junction proteins leading to elevation of paracellular permeability. The activation of NADPH oxidase leading to ROS generation following either TNF-α or IL-6 treatment of HBMvECs was also confirmed to be responsible in-part for these events. The overall outcome is a timely and wide-ranging human *in vitro* model of how different proinflammatory cytokines may potentially instigate BBB dysfunction during neurological diseases, and in our view, addresses many of the gaps and inconsistencies within the existing knowledge base. Extending this model to include other cytokines known to induce barrier dysfunction (e.g. IL-1 [50]) would further enhance our knowledge in this field. Moreover, whilst beyond the scope of the current study, a better understanding of the cytokine-mediated signaling events (common or otherwise) downstream of ROS generation, culminating in BBB downregulation, is of great interest. Cytokine-specific targeting of common proinflammatory transcription factors such as NF-κB leading to reduced gene expression [28], [40], [51] and/or ROS-mediated activation of the ubiquitin-proteasome system leading to enhanced protein turnover [52], [53] remain distinct possibilities.

## Supporting Information

Figure S1 **HBMvEC viability studies.** Confluent HBMvECs were stimulated with broad concentration ranges of (**A**) antioxidants - SOD (0–600 U/ml), CAT (0–1000 U/ml), NAC (0–30 mM), and APO (0–1 mM), and (**B**) cytokines – TNF-α (0–100 ng/ml) and IL-6 (0–100 ng/ml) for 18 hrs. Post-treatment, cells were harvested and prepared for viability assessment by flow cytometry. *P≤0.05 versus untreated control.
(TIF)

Figure S2 **Optimization of gp91 and p47 siRNA transfection in HBMvECs.** HBMvECs were transfected with gp91- and p47-specific siRNA (0–50 nM). Following cell recovery, whole cell protein lysates were harvested for Western blotting. Histograms represent the densitometric fold change in relative protein expression for gp91 (LHS) and p47 (RHS) in response to increasing concentrations of their respective siRNA. *P≤0.05 versus untransfected control. MT, mock transfection. All gels are representative.
(TIF)

Figure S3 **Dose-dependent effect of cytokines on interendothelial junction protein expression in HBMvECs.** Confluent cells were treated with TNF-α (**A**) or IL-6 (**B**) (0–100 ng/ml, 6 hrs). Post-treatment, whole cell protein lysates were harvested for Western blotting. Histograms represent the densitometric fold change in relative protein expression for VE-cadherin, occludin and claudin-5 (bars reading left to right) in response to increasing concentration of cytokine. *P≤0.05 versus untreated control. All gels are representative.
(TIF)

Figure S4 **Effect of ROS depleting agents on cytokine-induced ROS production in HBMvECs.** Confluent cells were pre-treated with either SOD (200 U/ml), CAT (200 U/ml), NAC (1 mM) or APO (10 µM), followed by treatment with TNF-α (**A**) or IL-6 (**B**) (100 ng/ml, 6 or 18 hrs). ROS production was subsequently monitored by flow cytometry using ROS-detecting DHE. Histograms (LHS) represent the fold change in fluorescent signal normalized to untreated control at 6 or 18 hrs. Representative FACS scans (RHS) are shown for both 6 and 18 hr treatments. Grey shaded scan indicates untreated control (full key beneath scans). *P≤0.05 versus untreated 6 or 18 hr controls. ^Ø^ *P*≤0.05 versus cytokine without ROS depleting agent.
(TIF)

Figure S5 **Effect of cytokines on NADPH oxidase activation in HBMvECs.** (**A**) Confluent cells were treated with TNF-α (LHS) or IL-6 (RHS) (0–100 ng/ml, 6 hrs) prior to harvesting of whole cell protein lysates for Western blotting. Histograms represent the densitometric fold change in relative protein expression for gp91 and p47 in response to increasing concentrations of cytokine. (**B**) Confluent cells were also treated with TNF-α (LHS) or IL-6 (RHS) (100 ng/ml, 6 hrs) prior to harvesting of whole cell protein lysates for co-IP. Histograms represent the densitometric fold change in gp91/p47 co-association in response to cytokine treatment. For each cytokine; LHS histogram = IP p47, WB gp91, RHS histogram = IP gp91, WB p47. *P≤0.05 versus untreated control. All gels are representative.
(TIF)

Figure S6 **Effect of NADPH oxidase blockade on cytokine-induced ROS generation in HBMvECs.** Confluent cells were either transfected with siRNA targeting gp91 or p47, or were pre-treated with NSC23776 (50 µM) for 1 hr prior to stimulation with TNF-α (**A**) or IL-6 (**B**) (100 ng/ml, 6 or 18 hrs). ROS generation was subsequently monitored by flow cytometry using ROS-detecting DHE. Histograms (LHS) represent the fold change in fluorescent signal normalised to untreated control at 6 or 18 hrs. Representative FACS scans (RHS) are shown for both 6 and 18 hr treatments. Grey shaded scan indicates untreated control (full key beneath scans). *P≤0.05 versus untreated control at 6 or 18 hrs. ^Ø^ *P*≤0.05 versus cytokine without NADPH oxidase blockade.
(TIF)

Figure S7 **Effect of antioxidant strategies on cytokine-induced HBMvEC barrier disruption.** (**A**) Confluent cells were treated with TNF-α (top) or IL-6 (bottom) (0–100 ng/ml, 6 hrs). Post-treatment, HBMvEC monolayer permeability was monitored by transendothelial permeability assay. Histograms represent the increase in % Transendothelial Exchange of FITC-Dextran 40 kDa (%TEE FD40) in response to increasing concentration of cytokine. (**B, C**) Prior to treatment with TNF-α (top) or IL-6 (bottom) (100 ng/ml, 6 hrs), confluent cells were pre-treated with either; (**B**) SOD (200 U/ml), CAT (200 U/ml), NAC (1 mM) or APO (10 µM); or (**C**) gp91 siRNA, p47 siRNA, or NSC23766 (50 mM). Following cytokine treatment, HBMvEC permeability was monitored. Histograms represent the change in permeability (%TEE FD40) in response to cytokines in the absence and presence of antioxidant treatment. *P≤0.05 versus untreated controls. ^Ø^ *P*≤0.05 versus cytokine without antioxidant treatment.
(TIF)

Figure S8 **Dose-dependent effect of cytokines on ZO-1 protein expression in HBMvECs.** Confluent cells were treated with TNF-α (LHS) or IL-6 (RHS) (0–100 ng/ml, 6 and 18 hrs). Post-treatment, whole cell protein lysates were harvested for Western blotting. Histograms represent the densitometric fold change in relative protein expression for ZO-1 in response to increasing concentration of cytokine. *P≤0.05 versus untreated control. All gels are representative.
(TIF)
