# Supplementary figures and images for: Downregulation of Blood-Brain Barrier Phenotype by Proinflammatory Cytokines Involves NADPH Oxidase-Dependent ROS Generation: Consequences for Interendothelial Adherens and Tight Junctions
Source: PLoS One. 2014 Jul 3;9(7):e101815. doi: 10.1371/journal.pone.0101815 (PMC4081725; doi:10.1371/journal.pone.0101815)

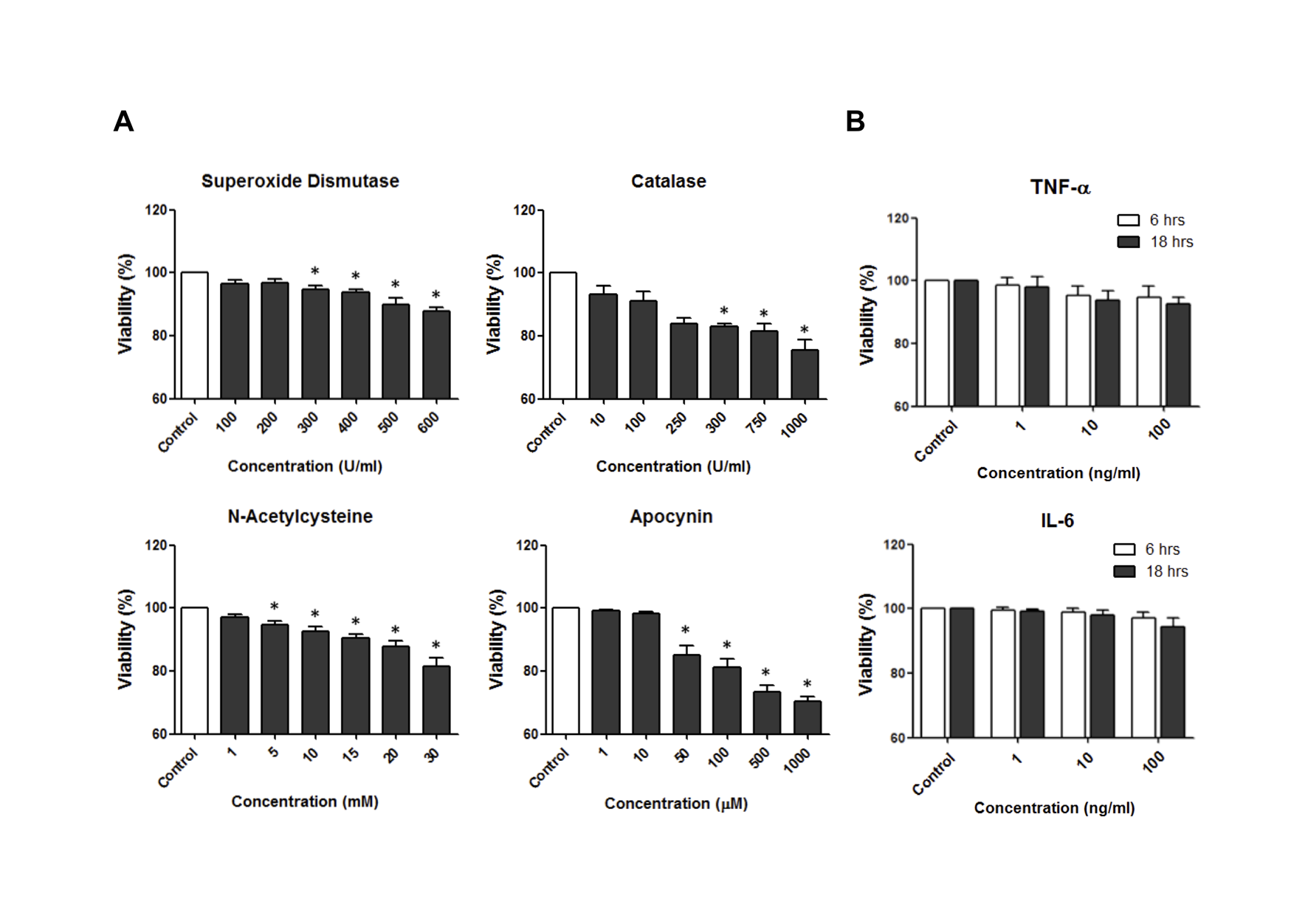

Supplement: Figure S1 — HBMvEC viability studies. Confluent HBMvECs were stimulated with broad concentration ranges of (A) antioxidants - SOD (0–600 U/ml), CAT (0–1000 U/ml), NAC (0–30 mM), and APO (0–1 mM), and (B) cytokines – TNF-α (0–100 ng/ml) and IL-6 (0–100 ng/ml) for 18 hrs. Post-treatment, cells were harvested and prepared for viability assessment by flow cytometry. *P≤0.05 versus untreated control. (TIF) [file pone.0101815.s001.tif]

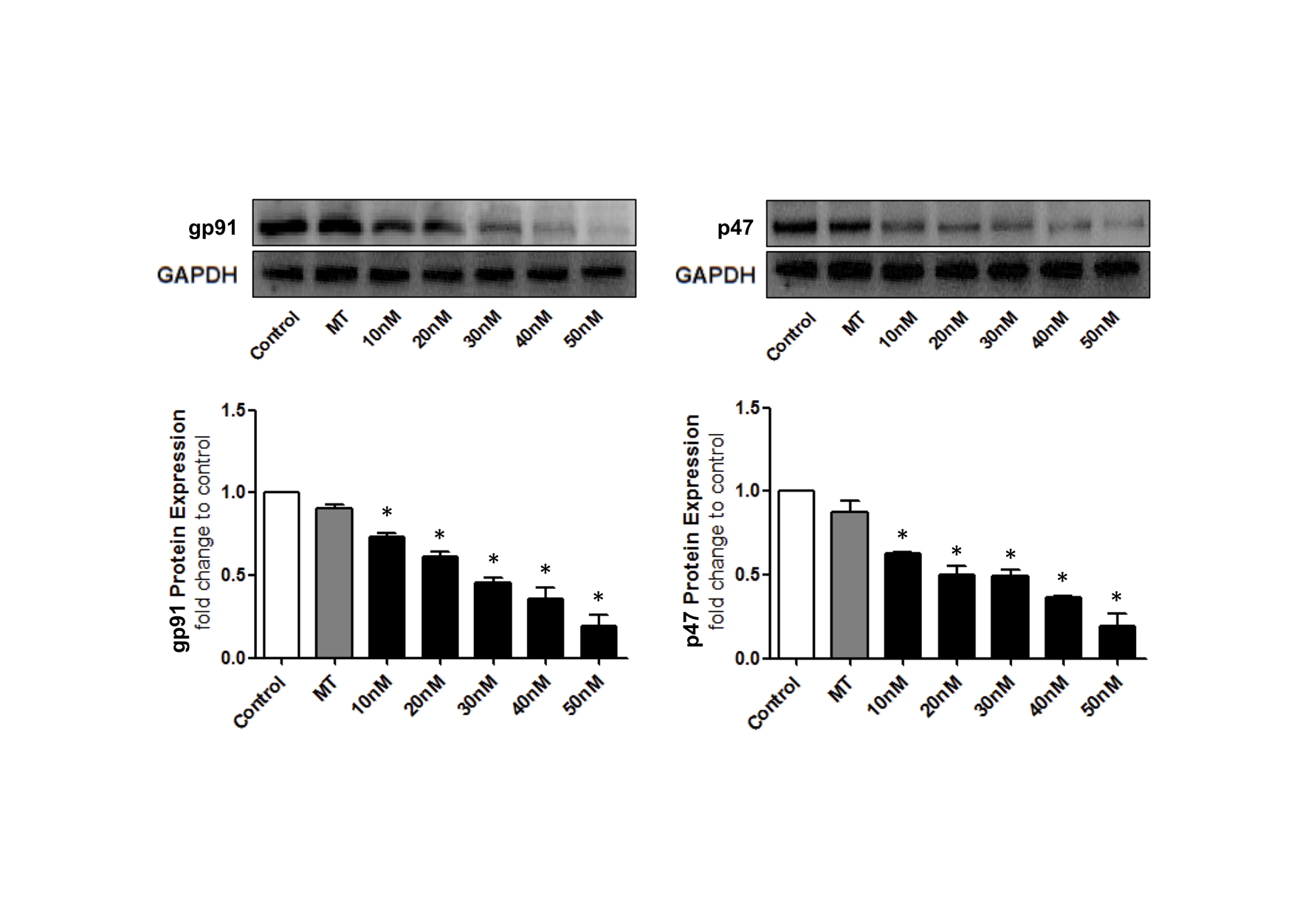

Supplement: Figure S2 — Optimization of gp91 and p47 siRNA transfection in HBMvECs. HBMvECs were transfected with gp91- and p47-specific siRNA (0–50 nM). Following cell recovery, whole cell protein lysates were harvested for Western blotting. Histograms represent the densitometric fold change in relative protein expression for gp91 (LHS) and p47 (RHS) in response to increasing concentrations of their respective siRNA. *P≤0.05 versus untransfected control. MT, mock transfection. All gels are representative. (TIF) [file pone.0101815.s002.tif]

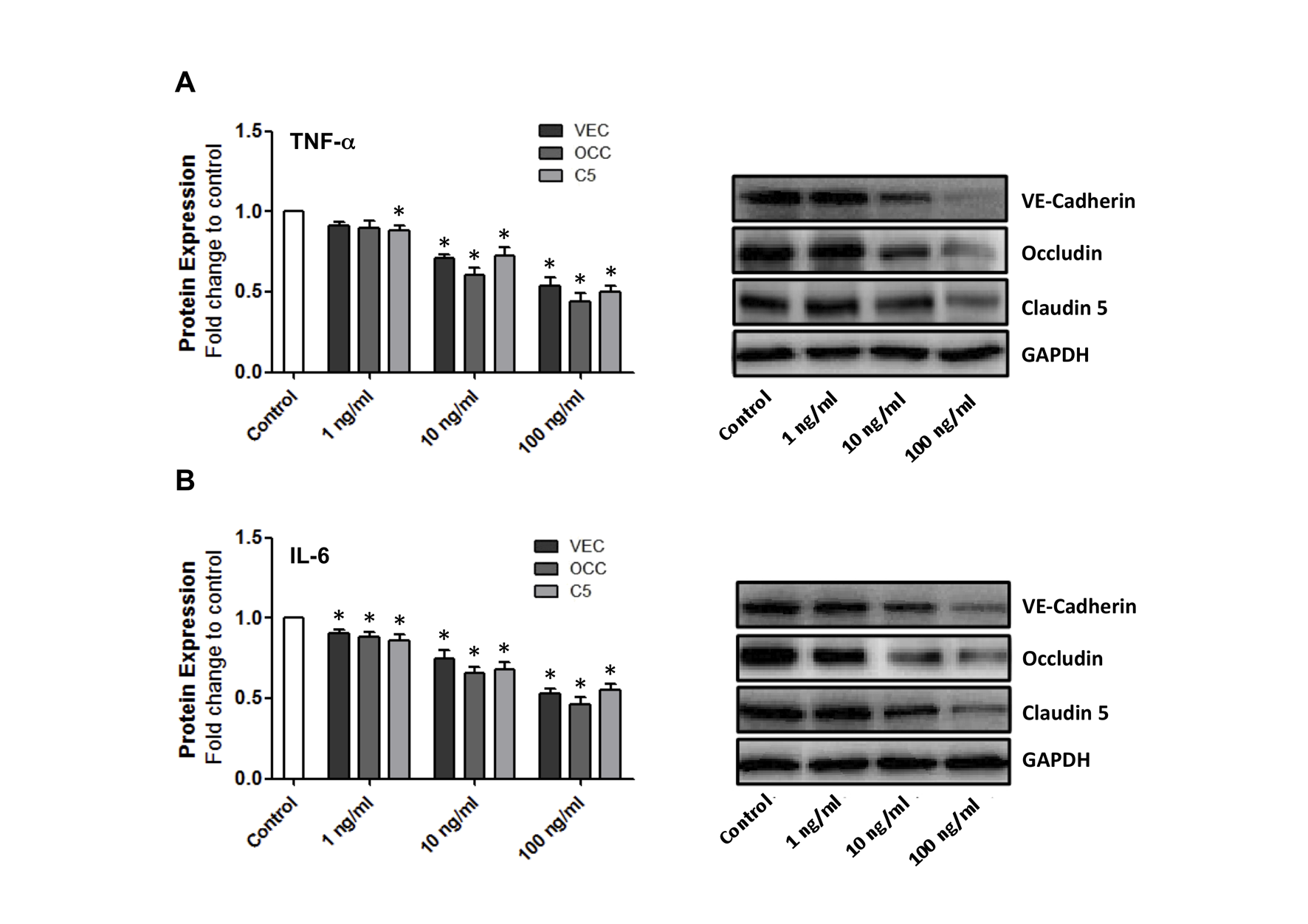

Supplement: Figure S3 — Dose-dependent effect of cytokines on interendothelial junction protein expression in HBMvECs. Confluent cells were treated with TNF-α (A) or IL-6 (B) (0–100 ng/ml, 6 hrs). Post-treatment, whole cell protein lysates were harvested for Western blotting. Histograms represent the densitometric fold change in relative protein expression for VE-cadherin, occludin and claudin-5 (bars reading left to right) in response to increasing concentration of cytokine. *P≤0.05 versus untreated control. All gels are representative. (TIF) [file pone.0101815.s003.tif]

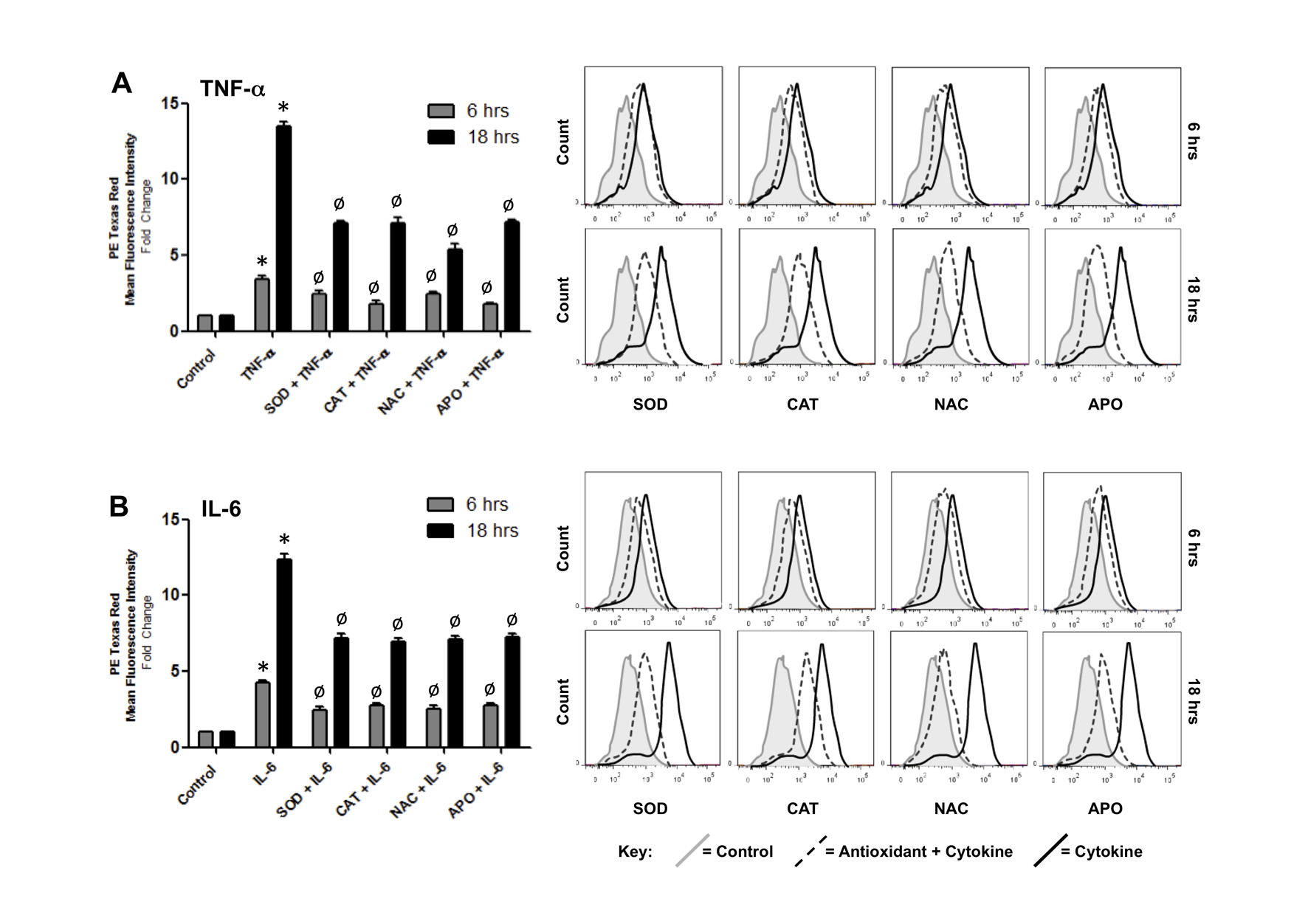

Supplement: Figure S4 — Effect of ROS depleting agents on cytokine-induced ROS production in HBMvECs. Confluent cells were pre-treated with either SOD (200 U/ml), CAT (200 U/ml), NAC (1 mM) or APO (10 µM), followed by treatment with TNF-α (A) or IL-6 (B) (100 ng/ml, 6 or 18 hrs). ROS production was subsequently monitored by flow cytometry using ROS-detecting DHE. Histograms (LHS) represent the fold change in fluorescent signal normalized to untreated control at 6 or 18 hrs. Representative FACS scans (RHS) are shown for both 6 and 18 hr treatments. Grey shaded scan indicates untreated control (full key beneath scans). *P≤0.05 versus untreated 6 or 18 hr controls. Ø P≤0.05 versus cytokine without ROS depleting agent. (TIF) [file pone.0101815.s004.tif]

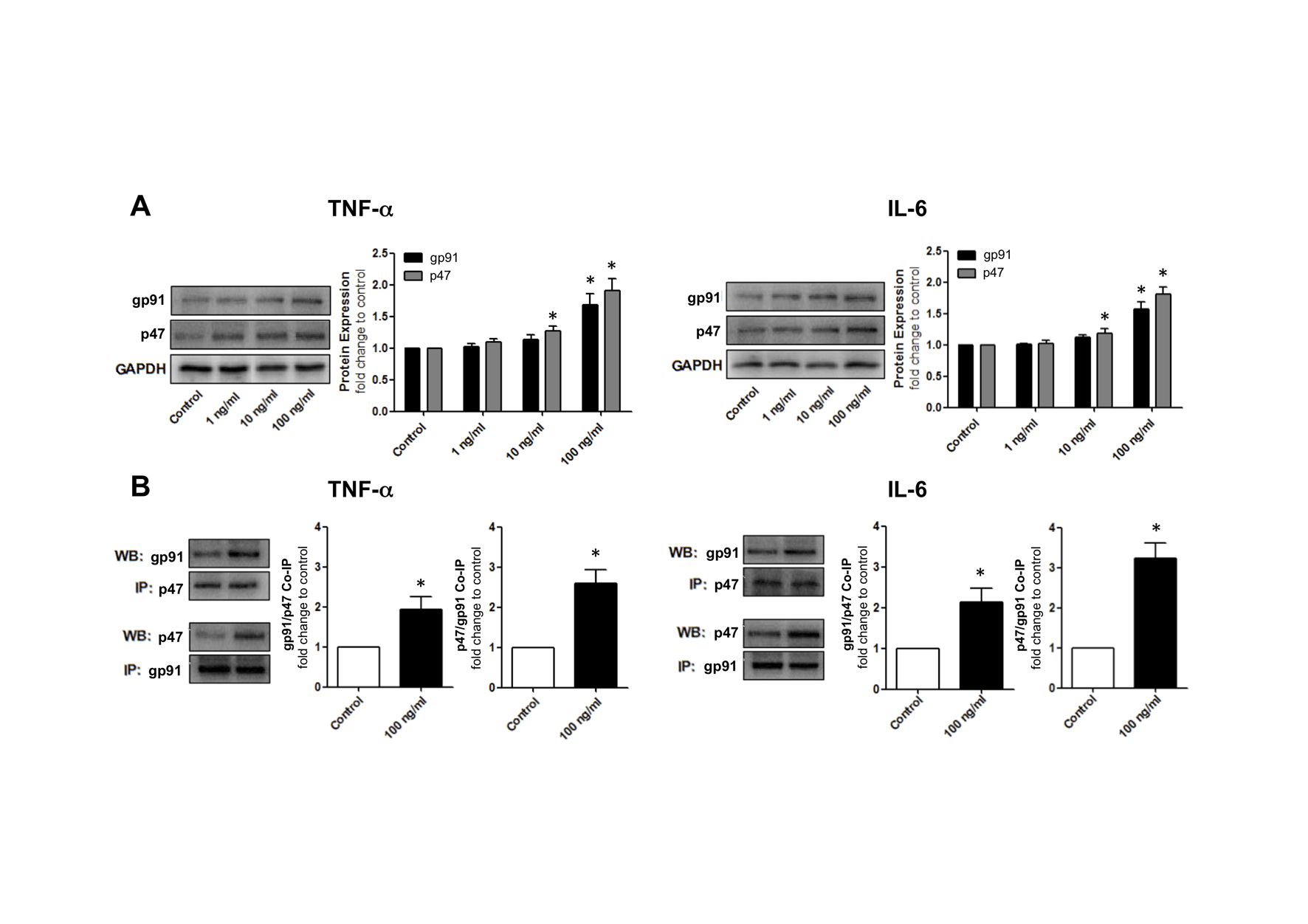

Supplement: Figure S5 — Effect of cytokines on NADPH oxidase activation in HBMvECs. (A) Confluent cells were treated with TNF-α (LHS) or IL-6 (RHS) (0–100 ng/ml, 6 hrs) prior to harvesting of whole cell protein lysates for Western blotting. Histograms represent the densitometric fold change in relative protein expression for gp91 and p47 in response to increasing concentrations of cytokine. (B) Confluent cells were also treated with TNF-α (LHS) or IL-6 (RHS) (100 ng/ml, 6 hrs) prior to harvesting of whole cell protein lysates for co-IP. Histograms represent the densitometric fold change in gp91/p47 co-association in response to cytokine treatment. For each cytokine; LHS histogram = IP p47, WB gp91, RHS histogram = IP gp91, WB p47. *P≤0.05 versus untreated control. All gels are representative. (TIF) [file pone.0101815.s005.tif]

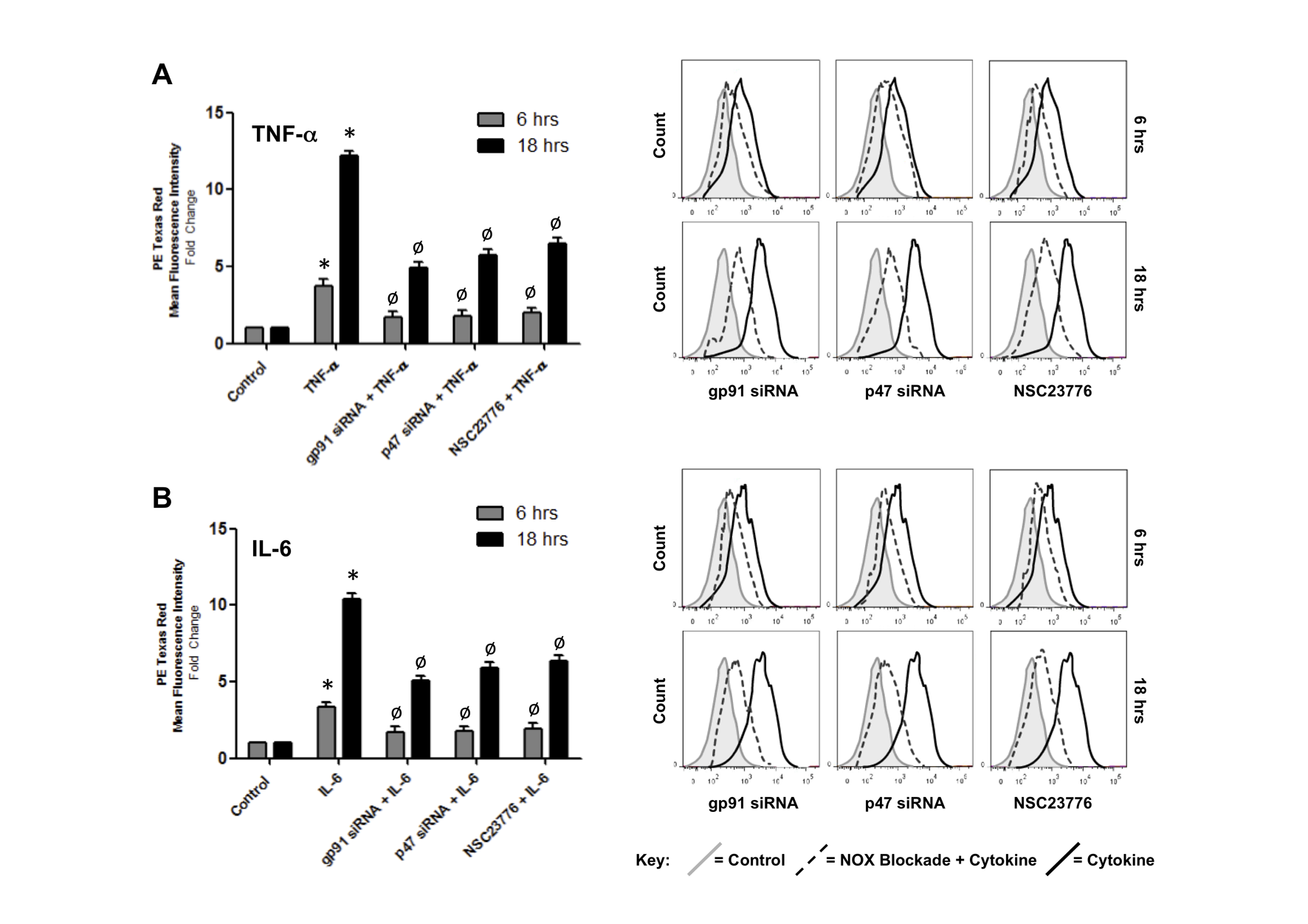

Supplement: Figure S6 — Effect of NADPH oxidase blockade on cytokine-induced ROS generation in HBMvECs. Confluent cells were either transfected with siRNA targeting gp91 or p47, or were pre-treated with NSC23776 (50 µM) for 1 hr prior to stimulation with TNF-α (A) or IL-6 (B) (100 ng/ml, 6 or 18 hrs). ROS generation was subsequently monitored by flow cytometry using ROS-detecting DHE. Histograms (LHS) represent the fold change in fluorescent signal normalised to untreated control at 6 or 18 hrs. Representative FACS scans (RHS) are shown for both 6 and 18 hr treatments. Grey shaded scan indicates untreated control (full key beneath scans). *P≤0.05 versus untreated control at 6 or 18 hrs. Ø P≤0.05 versus cytokine without NADPH oxidase blockade. (TIF) [file pone.0101815.s006.tif]

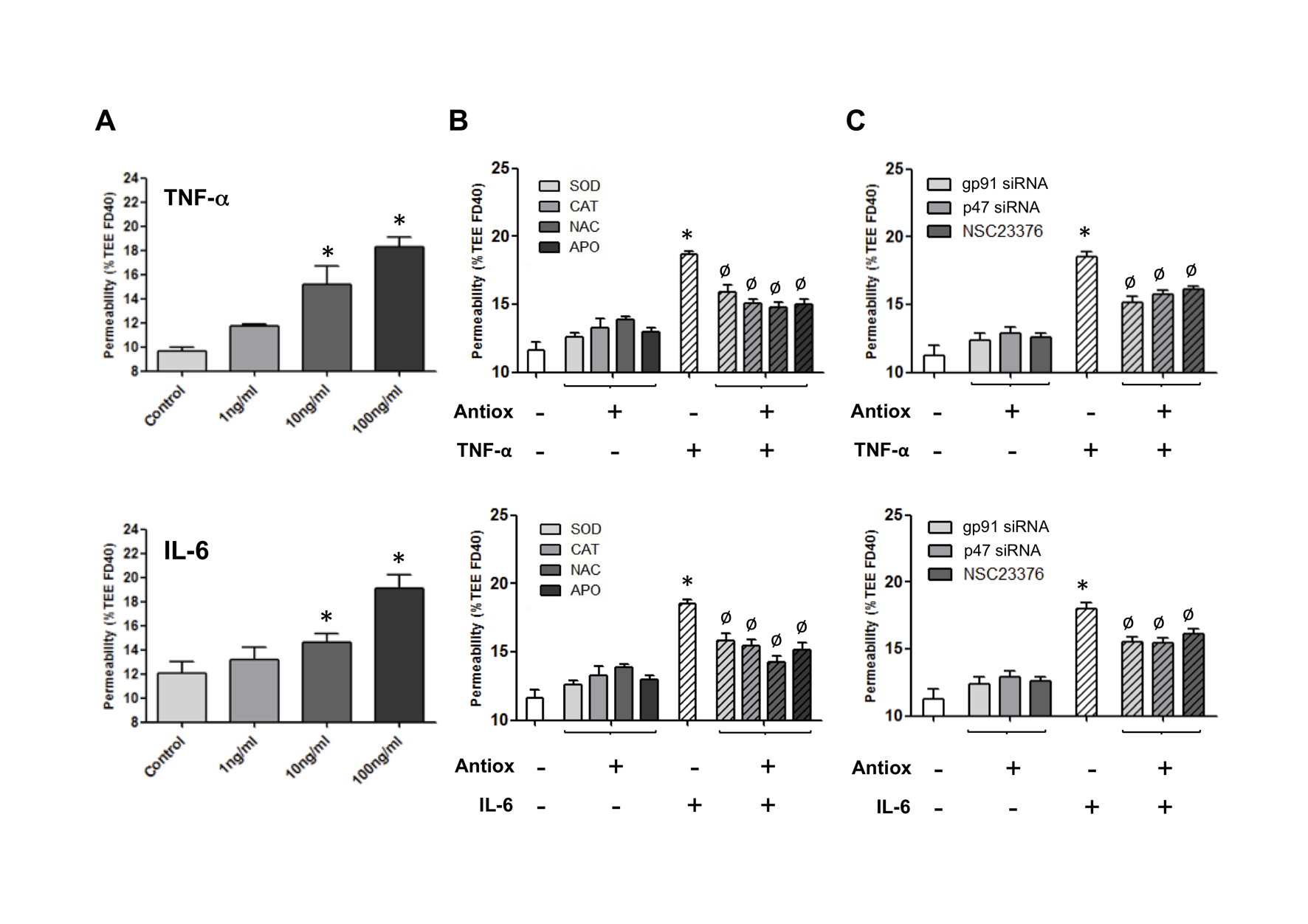

Supplement: Figure S7 — Effect of antioxidant strategies on cytokine-induced HBMvEC barrier disruption. (A) Confluent cells were treated with TNF-α (top) or IL-6 (bottom) (0–100 ng/ml, 6 hrs). Post-treatment, HBMvEC monolayer permeability was monitored by transendothelial permeability assay. Histograms represent the increase in % Transendothelial Exchange of FITC-Dextran 40 kDa (%TEE FD40) in response to increasing concentration of cytokine. (B, C) Prior to treatment with TNF-α (top) or IL-6 (bottom) (100 ng/ml, 6 hrs), confluent cells were pre-treated with either; (B) SOD (200 U/ml), CAT (200 U/ml), NAC (1 mM) or APO (10 µM); or (C) gp91 siRNA, p47 siRNA, or NSC23766 (50 mM). Following cytokine treatment, HBMvEC permeability was monitored. Histograms represent the change in permeability (%TEE FD40) in response to cytokines in the absence and presence of antioxidant treatment. *P≤0.05 versus untreated controls. Ø P≤0.05 versus cytokine without antioxidant treatment. (TIF) [file pone.0101815.s007.tif]

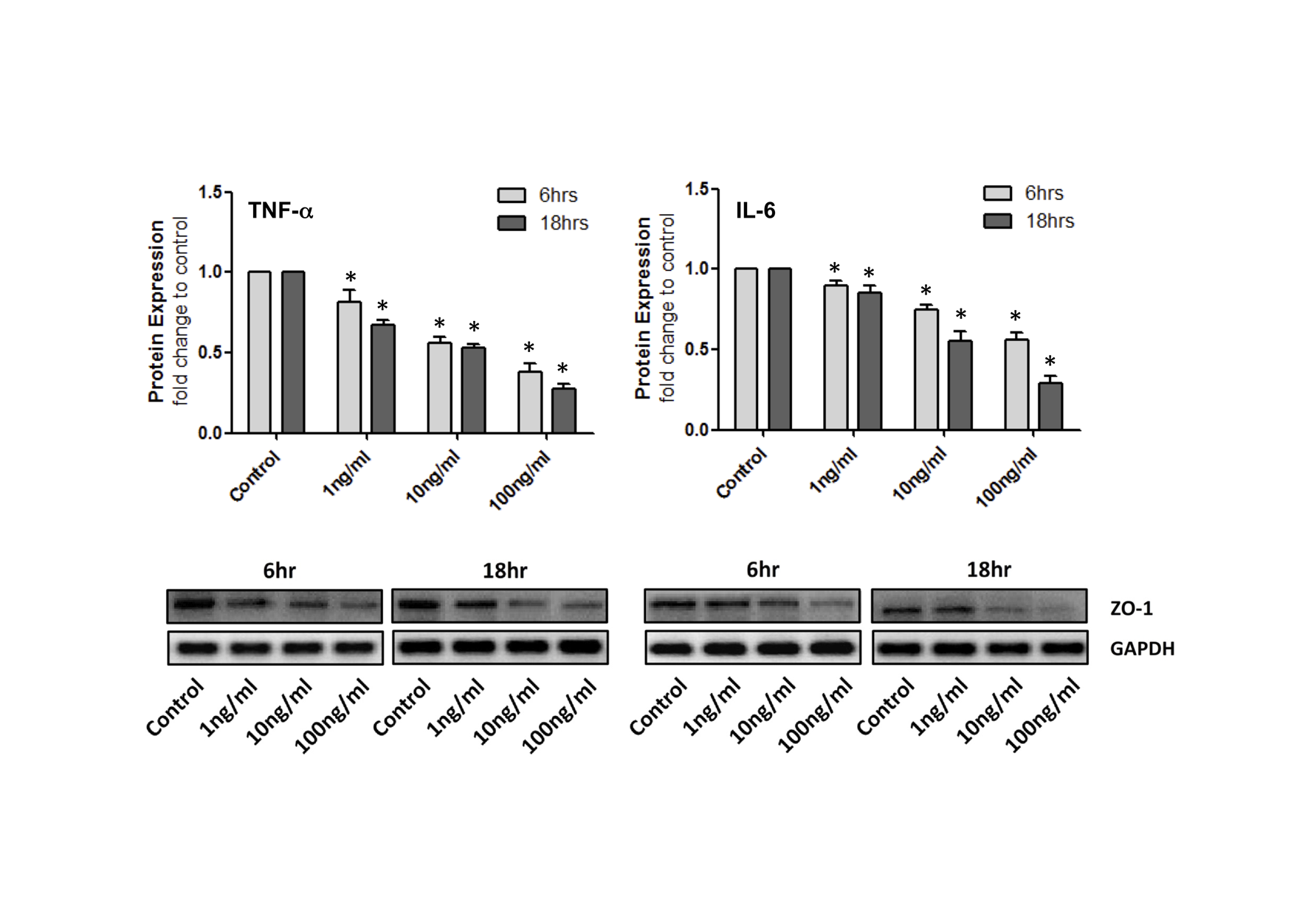

Supplement: Figure S8 — Dose-dependent effect of cytokines on ZO-1 protein expression in HBMvECs. Confluent cells were treated with TNF-α (LHS) or IL-6 (RHS) (0–100 ng/ml, 6 and 18 hrs). Post-treatment, whole cell protein lysates were harvested for Western blotting. Histograms represent the densitometric fold change in relative protein expression for ZO-1 in response to increasing concentration of cytokine. *P≤0.05 versus untreated control. All gels are representative. (TIF) [file pone.0101815.s008.tif]
